# Environmental Limits of Tall Shrubs in Alaska’s Arctic National Parks

**DOI:** 10.1371/journal.pone.0138387

**Published:** 2015-09-17

**Authors:** David K. Swanson

**Affiliations:** Arctic Inventory and Monitoring Network, National Park Service, Fairbanks, Alaska, United States of America; Chinese Academy of Sciences, CHINA

## Abstract

We sampled shrub canopy volume (height times area) and environmental factors (soil wetness, soil depth of thaw, soil pH, mean July air temperature, and typical date of spring snow loss) on 471 plots across five National Park Service units in northern Alaska. Our goal was to determine the environments where tall shrubs thrive and use this information to predict the location of future shrub expansion. The study area covers over 80,000 km^2^ and has mostly tundra vegetation. Large canopy volumes were uncommon, with volumes over 0.5 m^3^/m^2^ present on just 8% of plots. Shrub canopy volumes were highest where mean July temperatures were above 10.5°C and on weakly acid to neutral soils (pH of 6 to 7) with deep summer thaw (>80 cm) and good drainage. On many sites, flooding helped maintain favorable soil conditions for shrub growth. Canopy volumes were highest where the typical snow loss date was near 20 May; these represent sites that are neither strongly wind-scoured in the winter nor late to melt from deep snowdrifts. Individual species varied widely in the canopy volumes they attained and their response to the environmental factors. *Betula* sp. shrubs were the most common and quite tolerant of soil acidity, cold July temperatures, and shallow thaw depths, but they did not form high-volume canopies under these conditions. *Alnus viridis* formed the largest canopies and was tolerant of soil acidity down to about pH 5, but required more summer warmth (over 12°C) than the other species. The *Salix* species varied widely from *S*. *pulchra*, tolerant of wet and moderately acid soils, to *S*. *alaxensis*, requiring well-drained soils with near neutral pH. Nearly half of the land area in ARCN has mean July temperatures of 10.5 to 12.5°C, where 2°C of warming would bring temperatures into the range needed for all of the potential tall shrub species to form large canopies. However, limitations in the other environmental factors would probably prevent the formation of large shrub canopies on at least half of the land area with newly favorable temperatures after 2°C of warming.

## Introduction

**Fig 1:**
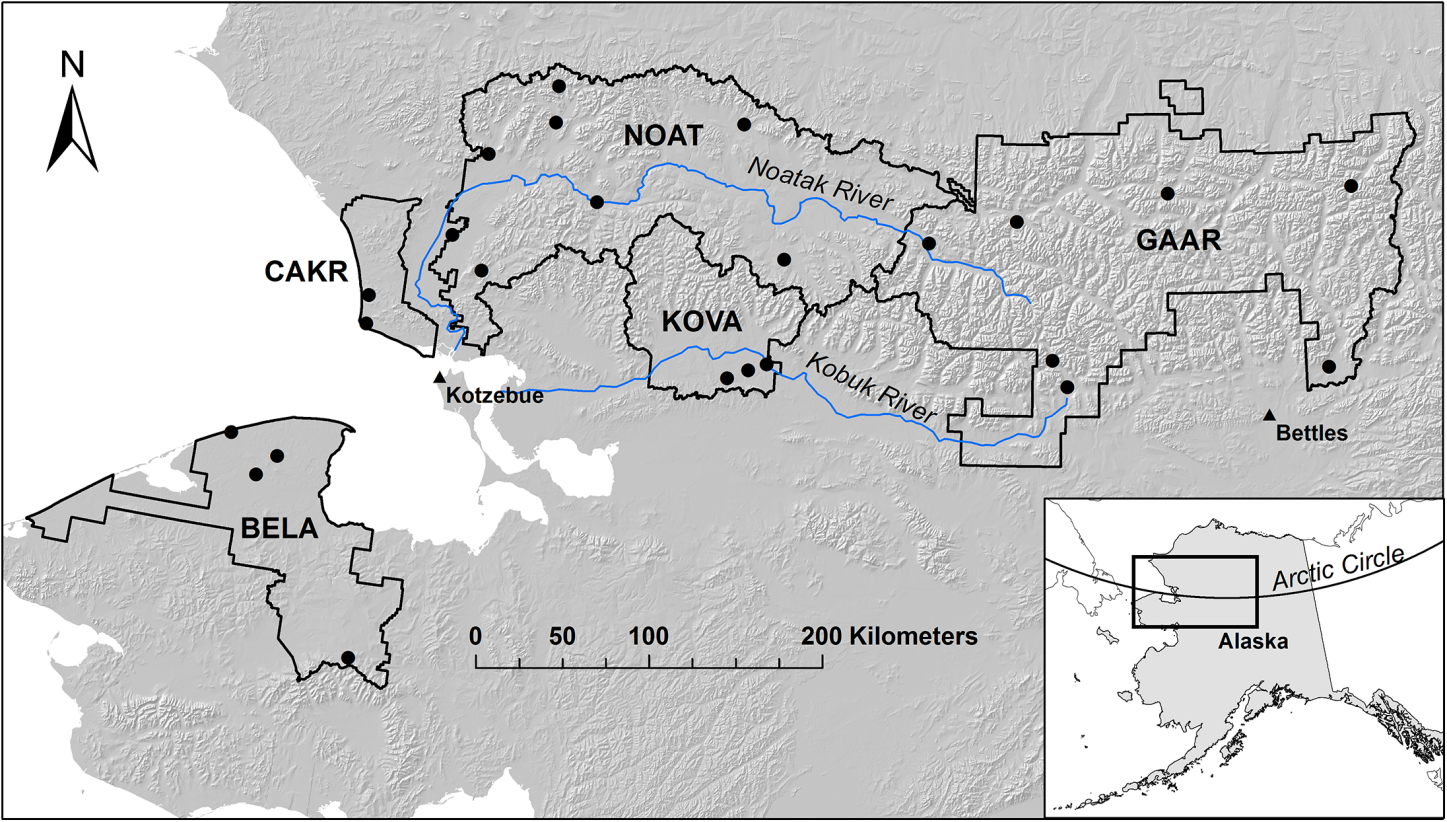
Study Area Location. The 5 National Park Service (NPS) units in northern Alaska make up the Arctic Inventory and Monitoring Network (ARCN). They are Bering Land Bridge National Preserve (BELA), Cape Krusenstern National Monument (CAKR), Gates of the Arctic National Park and Preserve (GAAR), Kobuk Valley National Park (KOVA), and Noatak National Preserve (NOAT). Sample nodes (n = 24, marked with black dots) are sample locations, each with approximately 20 sample plots.

Shrubs are the dominant plants over much of the arctic tundra [1]. Arctic shrubs range from tiny prostrate plants adapted to severe cold and wind [2], to tall shrubs that approach tree size near the boreal forest boundary. The most well-known landscape change in the Alaskan arctic in recent decades has been an increase in shrubs, probably as a result of climatic warming [3]. This shrub increase is visible by comparison of recent aerial photographs with historical photos taken in the 1950s and 1960s [4–7]. Shrub expansion appears to be a circumarctic phenomenon [4–6,8,9]. Warming-related shrub increases have also been observed in low-latitude alpine tundra [5,10]. We have documented an increase in tall shrubs in the United States National Park Service (NPS) Arctic Inventory and Monitoring Network (ARCN, the 5 NPS units in northern Alaska; Fig 1): an estimated 14% of the tall shrub vegetation in this region today has formed since about 1980 [11]. Shrubs provide forage for browsing species (moose, hares, and ptarmigan [12,13]). They also can reduce erosion by vegetating bare areas along streams [14], lead to greater fire frequency by increasing fuels [15], accelerate warming by decreasing albedo [16], increase snow depth by capturing windblown snow [8], and reduce plants of lower stature, such as lichens, by competition [17,18].

While warming-induced shrub expansion in the arctic is well documented, the future pace and proportion of the landscape affected by this vegetation change are difficult to predict. Global models of vegetation change in response to warming predict major biome shifts in the arctic, including migration of arctic treeline north across the study area, if plant dispersal is assumed to be unlimited [19,20]. In contrast, a state-and-transition model extrapolating recent rates of change into the future to year 2100 predicted relatively minor vegetation changes in the study area, the most important being loss of less than 10% of today's low shrub tundra by conversion to tall shrub or forest [21]. This dichotomy results from the fact that while just 2°C of warming would make low elevations in southern arctic regions warm enough to support tall shrubs or trees, the formation of these communities is both rate-limited (by plant dispersal and growth rates) and limited at a fine geographic scale to soils and sites favorable to the new communities. Analysis of the relationship between present-day tall shrubs and site conditions provides insight into the latter issue: how much of the landscape will have conditions suitable for tall shrubs in areas expected to become warm enough for tall shrubs in the near future?

During the summers of 2009 through 2014, NPS established a set of 471 vegetation monitoring plots in ARCN [22,23]. We collected data on height and cover by species of vascular and selected non-vascular plants on these plots, plus environmental data including a detailed soil description. Here I use these data to identify the environmental limitations on the abundance of tall shrubs in ARCN. I combine these results with spatial data to identify areas most susceptible to warming-induced future shrub expansion in the National Parks of northern Alaska. The shrubs species studied here have widespread distributions in North America and Eurasia, and the ecological tolerance information for these species presented in this paper should be useful elsewhere in the southern arctic tundra (bioclimate subzones D and E of [1]) of North America and Asia.

## Study Area and Methods

### Study Area

**Fig 2:**
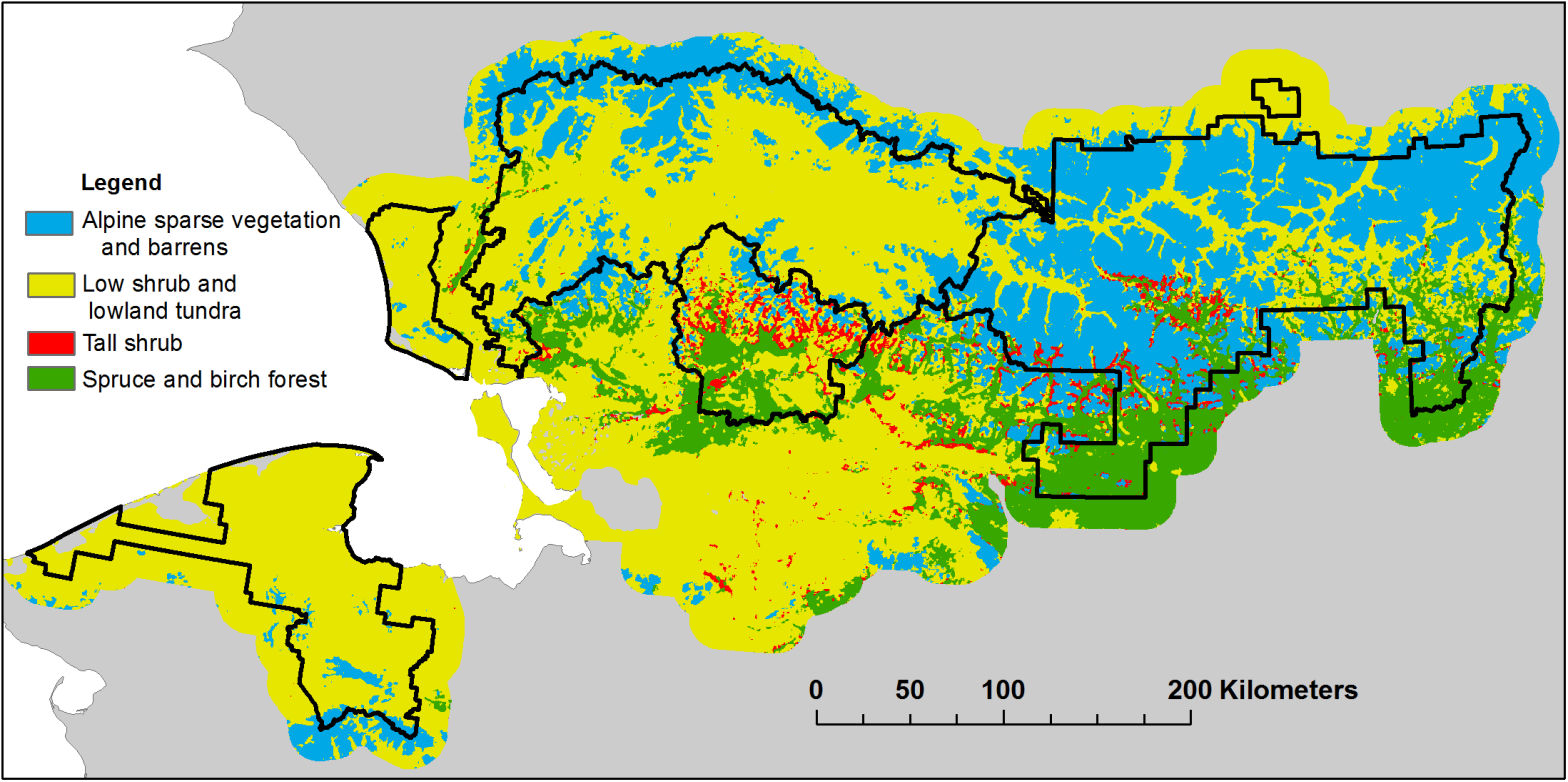
Vegetation Map of ARCN. Vegetation units are generalized from 44 types mapped by classification of satellite imagery [24].

**Fig 3:**
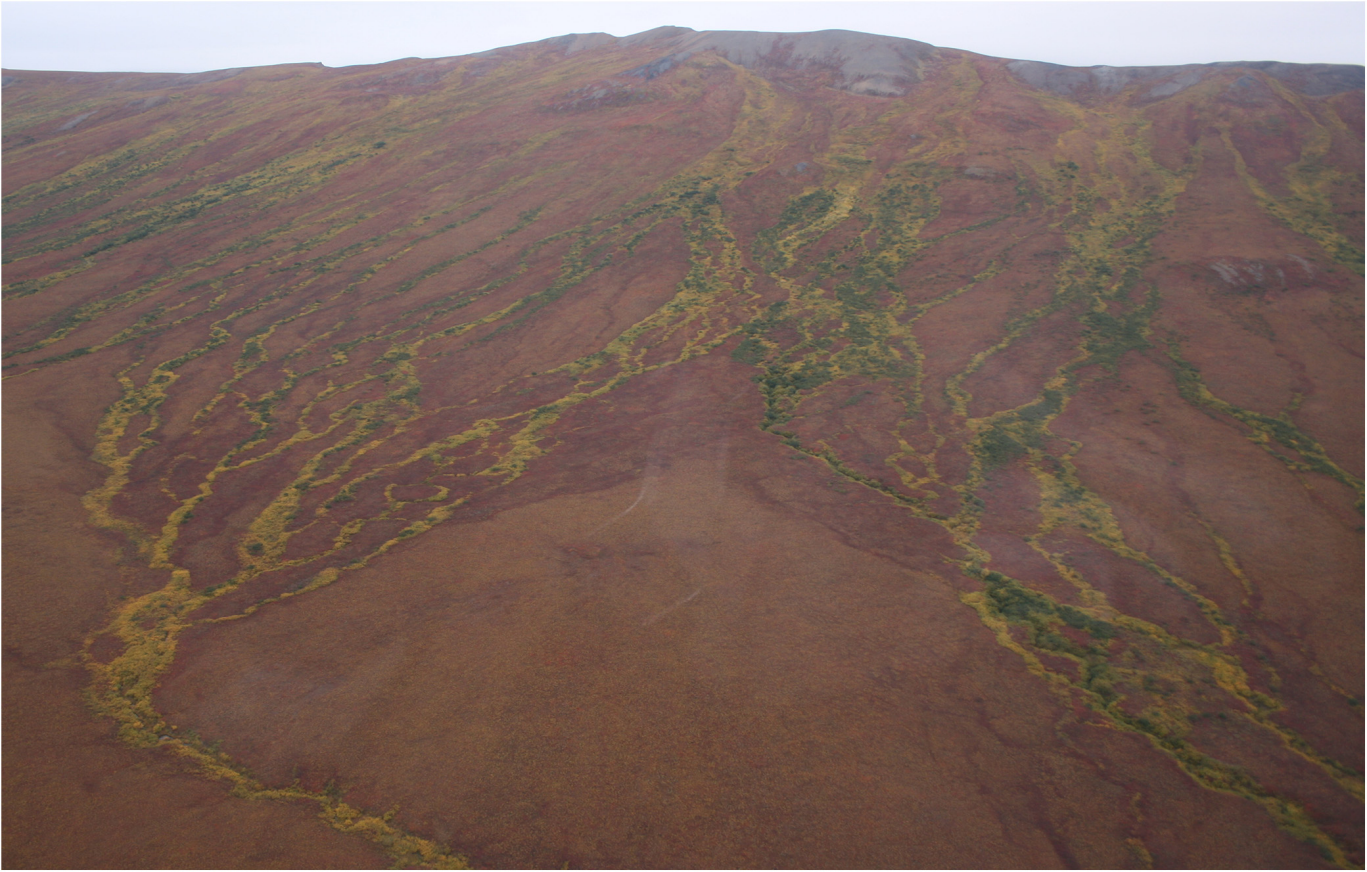
Shrub Thickets Along Tundra Drainageways. Elongated thickets of *Salix pulchra* (yellow) and alder (*Alnus viridis*, green areas) grow along wet drainageways in the Noatak National Preserve (27 August 2009). Areas between the drainageways support *Betula nana* (darker reddish areas mostly in the upper half of the photo), or tussock tundra with sparse shrub cover (brownish areas in the foreground).

**Fig 4:**
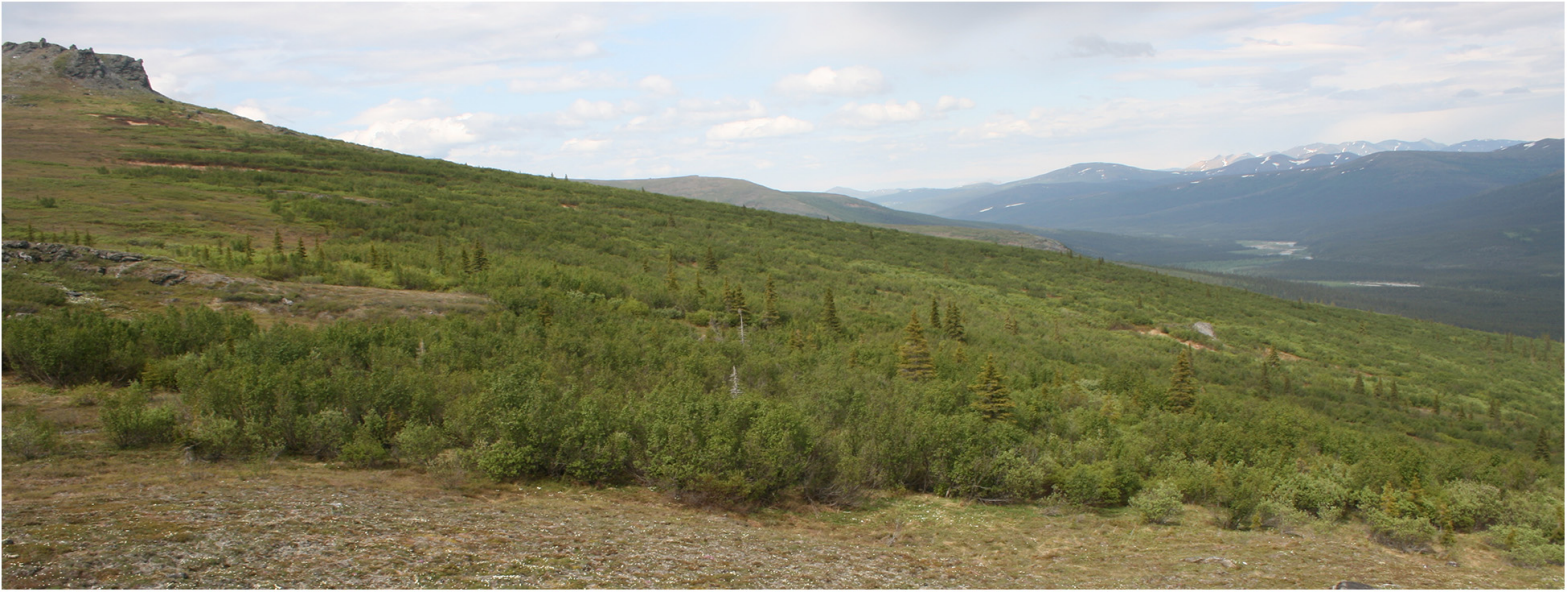
Tall Shrubs Near Treeline. Thickets of alder (*Alnus viridis*) dominate on well-drained soils just above treeline over parts of the south slope of the Brooks Range. Trees are white spruce (*Picea glauca*). Photo from Kobuk Valley National Park, 17 June 2009.

The study area (ARCN, Fig 1) is a mostly roadless area of over 8 million hectares, nearly one-fourth of the total land area nationwide managed by the NPS. Field permits were granted by the National Park Service: Gates of the Arctic National Park and Preserve, and the Western Arctic National Parklands. ARCN encompasses most of the central and western Brooks Range, with elevations up to about 2500 m, and adjacent lowlands down to sea level. The vegetation of ARCN consists mainly of arctic tundra, with boreal spruce and birch forest in the south at low elevations in the inland parks (Fig 2). Low shrub- and herb-dominated vegetation occurs on lowland tundra throughout the network, and in wetlands in the boreal forest to the south. Sparse alpine vegetation and barrens dominate at high elevations. Tall shrub communities are most common on floodplains, along drainageways on slopes, and near treeline (Figs 3 and 4). They occur in small patches at low to moderate elevations throughout the network, with only their most extensive occurrences shown in Fig 2.

**Fig 5:**
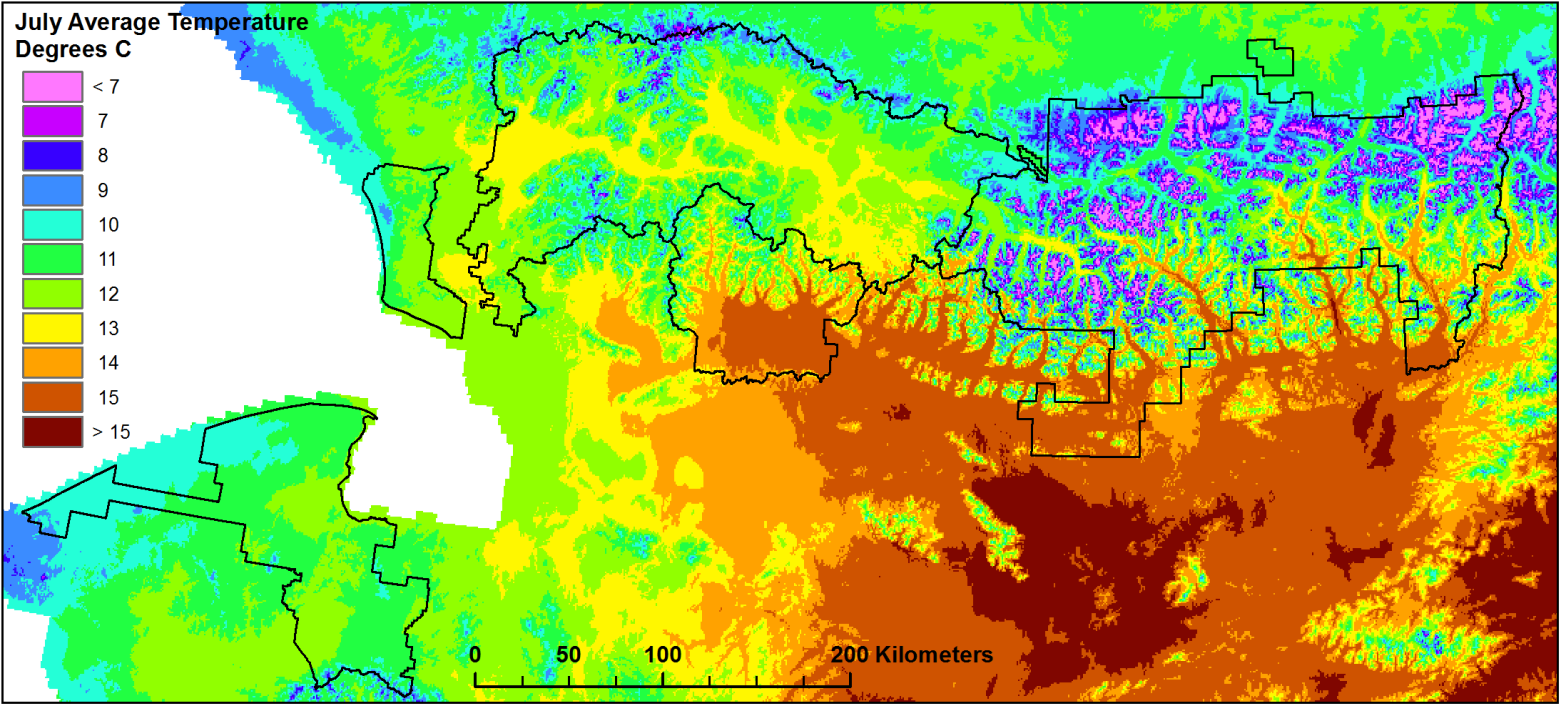
Mean July Temperature 1971–2000 in ARCN. Modeled values by PRISM Climate Group [25], rounded to the nearest integer.

A range of arctic and boreal climates are present in ARCN. Mean annual air temperatures range from about -5°C at low elevations across southern portions of all the parks to about -10°C at high elevations and along the northern edge of the NOAT and GAAR (climate data from modeling by PRISM Climate Group [25]; see Fig 1 for park unit abbreviations). January mean temperatures range from about -17°C in maritime areas in the west to about -26°C in the eastern Noatak Valley. Summers are warmest (July mean 15°C) in inland southern valleys, cooler in the maritime west (around 10°C) and coldest (below 5°C) high in the Brooks Range (Fig 5). Mean annual precipitation is 30–40 cm in lowlands of the west and south and 40 to 75 cm in the highlands; the monthly precipitation maximum occurs in August. Lowlands are dominantly wetlands and lakes, indicating a positive annual water balance, in spite of the low annual precipitation.

**Fig 6:**
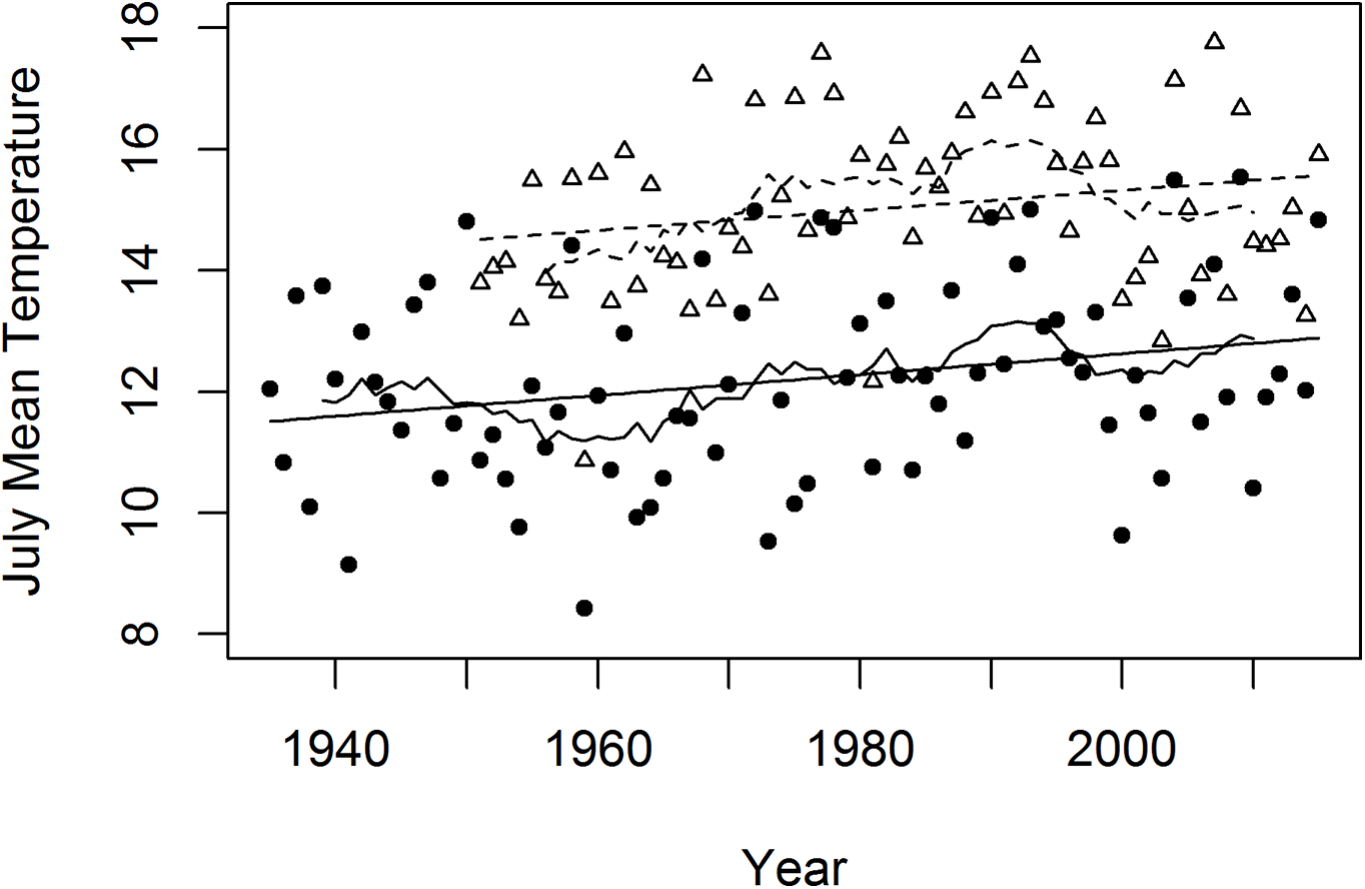
July Mean Temperatures at Kotzebue (1935–2015; circles) and Bettles (1951–2015; triangles), Alaska. The straight trendlines are linear regressions where x is the calendar year and y is mean July temperatures in °C: y = 0.01719x − 21.74, r^2^ = 0.06, P = 0.02 for Kotzebue (lower) and y = 0.01651X − 17.69, r^2^ = 0.05, P = 0.08 (upper) for Bettles. The regression slopes suggests an increase in July temperature of about 1.7°C per century at both stations. The irregular lines are 11-year moving averages for Kotzebue (lower) and Bettles (upper). Data from the Western Regional Climate Center [27].

Stafford et al. [26] reported an increase in mean annual temperature in northwestern Alaska over the period 1949–1998 of about 1.5°C. The greatest warming was in the winter, but summer temperatures increased by about 1°C. July mean temperatures at both Kotzebue and Bettles, the nearest stations with at least 50 years of data, increased at an average rate of about 1.7°C per century over their respective periods of record (Fig 6).

### Sample Design and Plots

Vegetation plots were clustered at 24 “nodes”, which consist of a set of approximately 20 plots (range: 11 to 29 plots) accessed from a central campsite (Fig 1; [22,23]). The node locations were chosen to be economical to access by air, and representative of major ARCN ecosystems and the full range of major environmental gradients. The vicinity of each node was stratified into landform-based physiographic units, and proposed transect locations were placed within each stratum. Transect starting points and azimuths were randomized, and plots were located systematically on the transects. A total of 471 plots were sampled. Because node and transect locations were located deliberately, the samples cannot be assumed to be in proportion to the area covered by the various environments in ARCN; in fact, we deliberately targeted some ecosystems with small areas, such as floodplains, to ensure coverage of the full range of environmental conditions present. The result is a data set that spans the range of major environmental gradients: elevation, continentality (east-west distance from the ocean), vegetation from boreal forest to alpine barrens, calcareous and non-calcareous substrates, and wet to dry soils. The clustering of plot locations allowed us to greatly increase our sample size over what would have been possible with isolated random or systematic points.

At each plot, plant cover and height by species (or species group for many non-vascular plants) were measured by point intercept using a laser at 100 points spaced 25 cm apart on two lines; the lines were 16 m long each and perpendicular, intersecting in the middle, with no points in the middle 4 m of each line. The name and height class (Table 1) of the uppermost intercept of each vascular plant species was recorded at each point.

**Table 1:** Point-Intercept Height Classes

Height Class, cm	Representative Height, cm^a^
0–20	10
20–50	35
50–100	75
100–150	125
150–300	225
over 300	400

^a^The midpoint value for the height class, used to compute shrub canopy volume. The 400 cm value for the “over 300 cm” height class was chosen because tall shrubs rarely exceed 5 m in our study area.

A soil pit was placed at the plot center near where the point-intercept lines intersect. The soil was described by standard US Department of Agriculture methods [28,29]. Soils were described to a depth of about 75 cm, or less if digging was prevented by frozen soil or rock. The description was continued by bucket auger to a depth of 1.5 m where possible. The soil pH was measured for each genetic horizon using the Morgan Method of multiple liquid field indicators, with total range of 3.8 to 8.4 and resolution of 0.2 units [30].

Soil descriptions are missing from one node (the Cape Krusenstern node in CAKR, 12 plots) where concern for archeological resources precluded digging soil pits, and from 10 other plots where soil was absent on talus or rock.

### Shrub Canopy Volume

All shrub species that were recorded with a height class of 1 m or higher somewhere in our data set were analyzed in this study. These 12 species (and one hybrid) are referred to below as "potential tall shrub species". The effects of environmental factors on individual species were analyzed for the 7 most abundant of the potential tall shrub species: *Alnus viridis* (36 plots), *Betula glandulosa* plus *B*. *nana* (247 plots), *Salix alaxensis* (36), *Salix glauca* (48), *Salix pulchra* (121), and *Salix richardsonii* (46). *B*. *glandulosa* and *B*. *nana* were combined in this study because they intergrade in our study area, and individual plants frequently cannot be assigned with certainty to one species. The remaining five, less common potential tall shrub species and the hybrid were included in the analysis of the total shrub canopy, but these species were not analyzed individually: *Salix arbusculoides* (4 plots), *Salix bebbiana* (2 plots), *Salix hastata* (16), *Salix niphoclada* (9), *Salix pseudomonticola* (4), and hybrid shrub-tree birches (*Betula glandulosa* or *B*. *nana* x *B*. *neoalaskana*, 3 plots).

Each point-intercept observation of the shrub species listed was given the representative height for its height class (Table 1). These representative heights were summed for the plot and divided by 100 (the number of point observations per plot). The result is an estimate of average shrub canopy volume, as expressed in cubic meters of canopy per square meter of ground, which reduces to meters. The canopy volume in meters can be visualized as the height of the canopy if the volume were evenly distributed across the whole area. For example, 50 hits in the 1–1.5 m height class (representative height of 1.25 m) out of 100 points yields a volume of 0.625 m. The shrub canopy volume was computed for the selected individual species, and for the total tall shrub canopy. The latter aggregated all of the potential tall shrub species listed above. Where two or more tall shrub species were encountered at a single point, the hit on the highest shrub at the point was used to compute the total shrub canopy volume.

### Environmental Factors

The volume of the shrub canopy was studied in relation to soil summer depth of thaw, soil drainage (wetness), soil acidity, normal date of loss of snow cover in the spring, and mean July air temperature. These factors were chosen because they express the fundamental site conditions of warmth, water, and nutrient supply. The date of snow loss provides additional information about winter wind abrasion and desiccation of plants and shortening of the growing season by deep snow [8]. The disturbance processes of flooding and wildfire were also assessed.

#### Soil depth of thaw

Soil depth of thaw was recorded on the day of sampling: between 1 July and 20 August with an average sample date of July 20. Because the depth of thaw increases throughout the summer, the observed depth of thaw in each soil was normalized to an estimated July 20 value. Note that this is not the depth to permafrost, which would be the maximum depth of thaw and is typically recorded in September. The July 20 depth of thaw is both more accurately estimated (since most samples were made within 2 weeks of this date) and more relevant to plant growth, since July is the middle of the growing season. Plant senescence begins in mid-August in our study area, and thus any soil layer that becomes available by thaw after this time is not likely to contribute to plant growth.

The normalized thaw depths were computed as follows. The depth of thaw in a soil on any given day is proportional to the square root of the thaw season’s cumulative sum of thaw degree-days [31,32]. Thus the sum of thaw degree-days can be used to compute what proportion of the yearly thaw has occurred by a given date. Since our climate stations are widely spaced and far from our sample sites and data were collected across several years, I chose to compute an average correction factor for the entire study area. Average daily temperature data for the 4 climate stations with long-term data (at least 20 years: the Noatak, HooDoo, Kavet, and Kelly RAWS; [33]) were used to compute an average sum of thaw degree-days for each station and date. The square root of this value was computed for each date and then all values were divided by the July 20 value. The result was an estimate of the amount of thaw that typically occurs by each date, expressed as a proportion of the July 20th depth of thaw. These proportions ranged across the 4 stations from 0.78 to 0.82 on July 1, to 1.20 to 1.23 on August 20. The mean proportion was computed from the 4 stations and its reciprocal used to correct the observed thaw depths. The correction factor ranged from 1.25 for July 1 thaw depths to 0.82 for August 20 thaw depths.

These thaw depth were then converted to classes to allow for the soils with no frozen soil observed:

Class 1: adjusted depth to frozen soil less than 40 cm (124 plots)

Class 2: 41–60 cm (68 plots)

Class 3: 61–80 cm (18 plots)

Class 4: more than 80 cm or frozen soil not present (249 plots)

#### Soil drainage

Soil wetness was summarized by placing each plot into one of 5 soil drainage classes, following standard Soil Survey Manual practices as adapted for use in Alaska [28,34].

Class 1: Very Poorly Drained. Organic surface horizon was more than 40 cm thick, or at least 20 cm thick and composed the entire soil profile down to frozen material. Water was usually present within 30 cm of the soil surface at the time of sampling (July–August). Slope 0 to 2% (60 plots).

Class 2: Poorly Drained. Surface organic horizon less than 40 cm thick. Mineral soil had dominantly grayish colors due to reduced iron within 50 cm of the soil surface. Free water or saturated fine-grained soil was usually present within 40 cm of the surface during sampling (July–August). Also included soils composed of organic material down to frozen soil with slope greater than 2% (182 plots).

Class 3: Somewhat Poorly Drained. Surface organic horizon was less than 20 cm thick, and grayish colors due to reduced iron were present between 50 and 75 cm depth. This class is rare in the study area but was retained because it differs substantially from either neighboring class and has the potential to increase in the future due to permafrost thaw [35] (4 plots).

Class 4: Well to Moderately Well Drained. Surface organic horizon less than 20 cm thick. No grayish colors due to reduced iron, or brightly colored mottles due to iron migration and oxidation, were present. Free water or saturated soil was rarely present. Saturated hydraulic conductivity was “moderately high” or less as defined by the Soil Survey Manual (less than 10 μm/s) and estimated from field soil textures; these were loamy soil textures, excluding sandy loam textures with coarse fragments greater than 35% by volume. Slopes were less than 30% (69 plots).

Class 5: Excessively to Somewhat Excessively Drained. Like class 4 except saturated hydraulic conductivity in the “high” class as defined by the Soil Survey Manual (10–100 μm/s) and estimated from field soil textures; these were sand or loamy sand textures, or sandy loam textures with coarse fragments greater than 35% by volume. Also included were soils with properties like class 4 but with slope greater than 30% (144 plots).

#### Soil acidity

**Fig 7:**
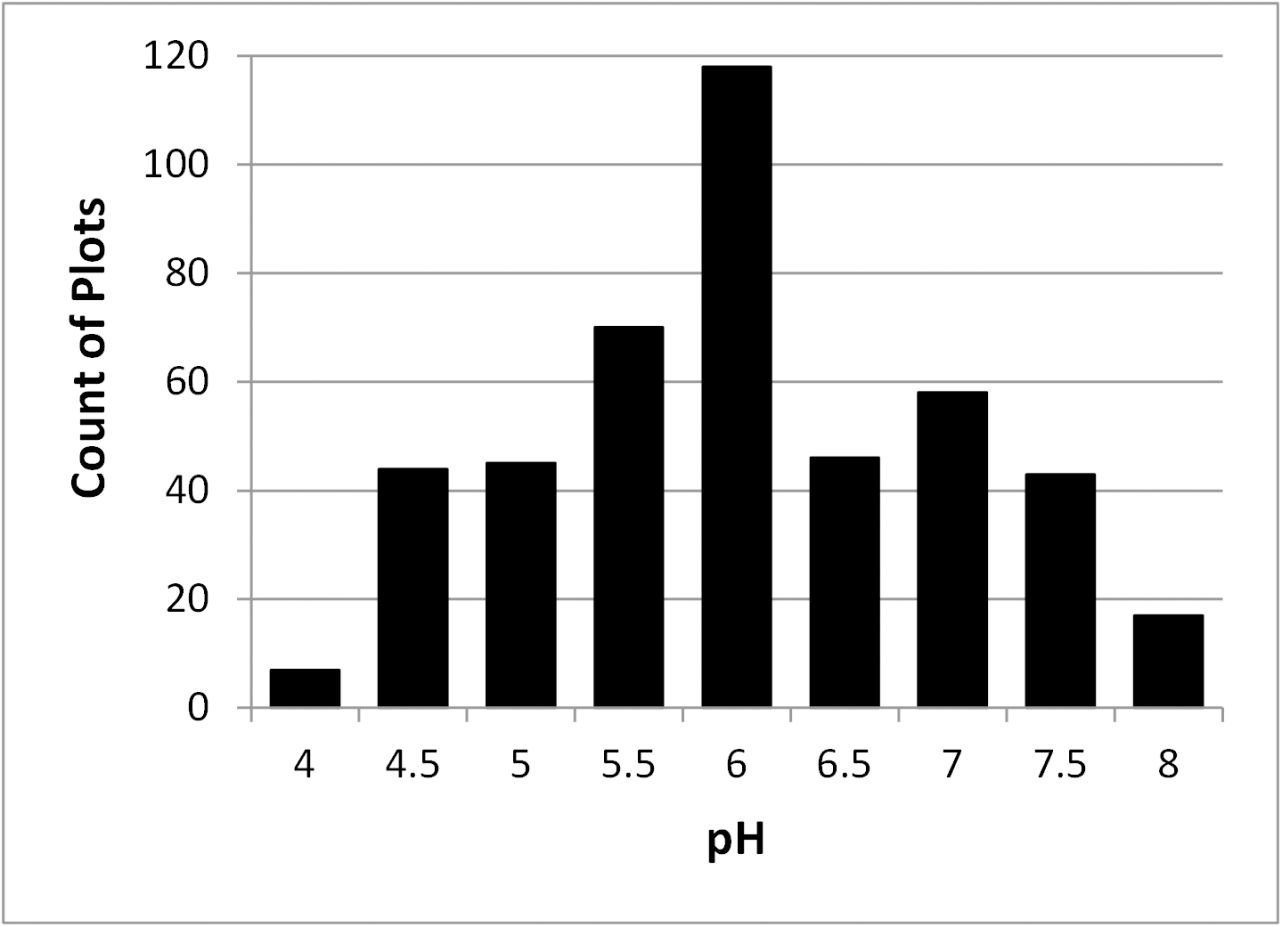
Distribution of weighted average rooting zone soil pH values. The count of plots is given for classes, corresponding to pH rounded to the nearest 0.5 units.

Because pH can vary markedly between horizons in a soil profile, a weighted average was computed for the rooting zone. The soil horizon root abundance classes from the field soil descriptions (few, common, and many; [28]) were assigned numerical values of 1, 2, and 3 respectively. Root abundance was estimated for all roots, not separately for shrubs as they not separable in most cases. The pH of a horizon was then weighted by the root abundance and the horizon thickness, and the weighted average pH was computed for the entire rooting zone. Weighted average pH values were available for 449 plots and ranged from 3.9 to 8.2 (Fig 7).

#### Normal date of snow cover loss

**Fig 8:**
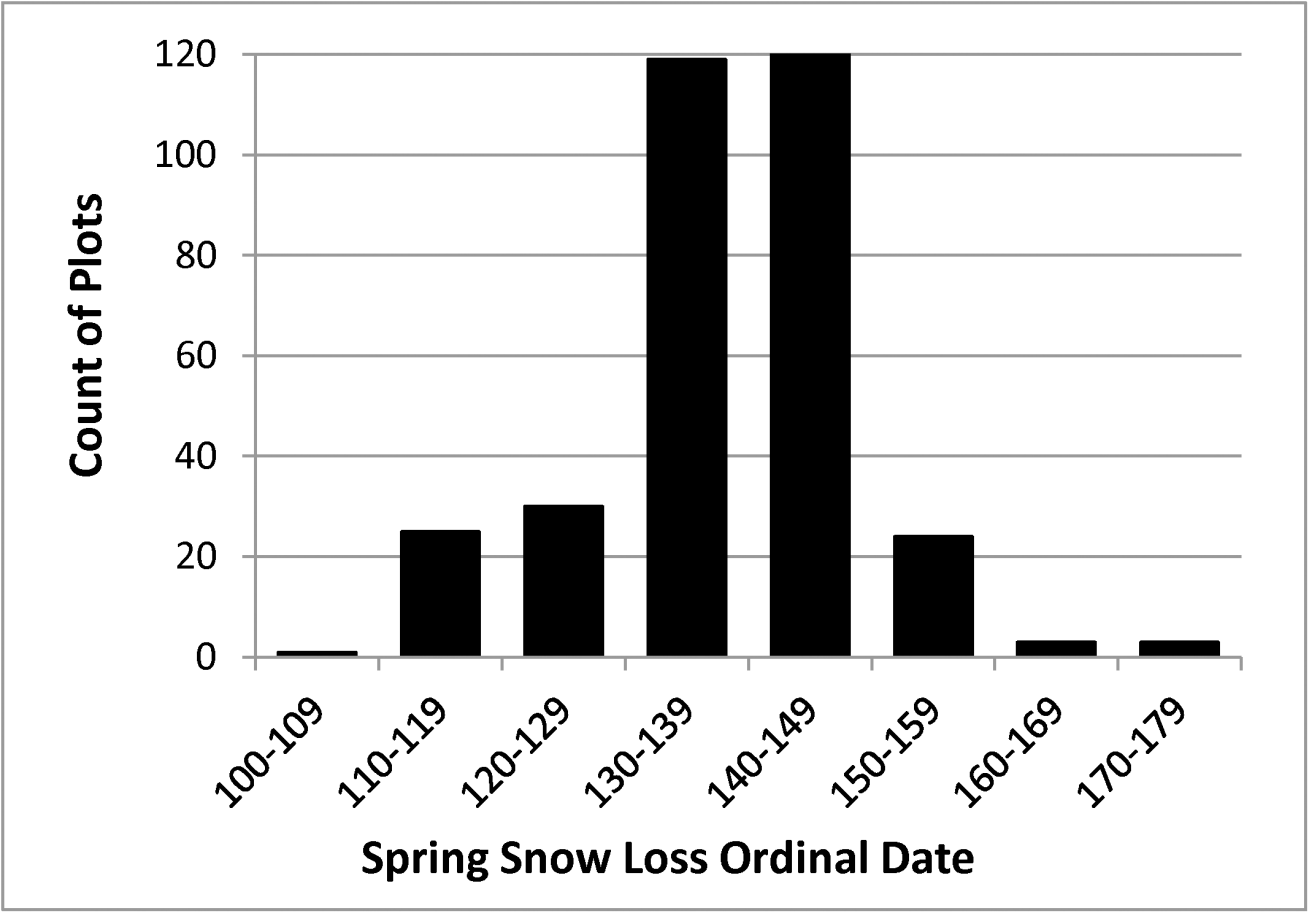
Normal Date of Snow Cover Loss on the Sample Plots. Snow loss ordinal dates are from Macander and Swingley's[36] analysis of Landsat data for the period 1985–2011.

The normal date of snow cover loss was extracted by overlay of the plot coordinates on a raster map of normal snow loss date by Macander and Swingley ([36]; 30 m pixel resolution). This map was produced by classification of Landsat data for the period 1985–2011 using a decision tree algorithm to select the date that best separated snow-covered and snow-free dates for each pixel. The date of snow cover loss identifies locations where late-lying snowbanks inhibit plant growth. It can also be used to identify areas where high winds may limit tall shrub growth. The thaw season in ARCN begins in late April or May at most elevations; snow cover is lost in ARCN prior to May 10 (day 130) primarily in places where the snowpack is thin or absent due to wind scour, or in places too steep to retain snow [37]. Snow-off dates of our plots ranged from day 107 to day 178 (17 April to June 27; Fig 8).

#### Mean July air temperature

The mean July air temperature was estimated by overlay of the plot coordinates on the raster map of mean 1971–2000 July temperature by the PRISM Climate Group ([25], 770 m resolution; Fig 5). The PRISM process uses available climate station data and interpolates a complete climate grid using a peer-reviewed model that incorporates location, topography, and other variables [38]. Estimated mean July temperatures at the 471 sample plots ranged from 7.6°C to 15.2°C. The mean July temperature is, of course, closely related to the mean summer (June, July, and August) temperature, which covers essentially the entire growing season in our climate. For the PRISM data set the mean summer temperature averages 1.8°C lower than the mean July temperature, with a standard deviation of 0.24°C.

Independent data from 9 climate stations on a north-south transect in northern Alaska just east of the study area during the model time period (1971–2000) provide a picture of the accuracy of the PRISM modeled temperatures [39]. The mean square difference between the modeled and observed mean July temperatures at the nine stations was 0.3°C, and the mean absolute difference was 0°C. This suggests that errors are minor and not biased in one direction, though it is still possible that some systematic bias is present in other parts of the study area.

#### Disturbance

The main disturbance processes operating on our sample plots are wildfires and alluvial flooding. Wildfire-affected plots were identified by field observations (in the case of one set of plots that had burned the previous year) and mapped historical fire perimeters, which are available from the present back to 1940 [40]. Plots affected by flooding were identified by a combination of alluvial sediments and evidence of alluvial deposition (buried soil horizons) in the soil pit, alluvial landforms, and proximity to a stream.

#### Interdependence of the environmental factors

Soil drainage and soil thaw depth are closely linked; areas with deep thaw depths usually have good drainage, and soils with shallow thaw have poor or very poor drainage (Table 2). However, I analyzed shrubs in relation to both thaw depth class and drainage class because they retain some important independent information: poor drainage can occur with any thaw depth.

**Table 2:** Correspondence of Soil Drainage and Soil Thaw Depth Classes^a^.

Soil Drainage Class	Count of Plots by Soil Thaw Depth Class				Total
	1: < 40 cm	2: 40–60 cm	3: 60–80 cm	4: >80 cm	
**1: very poor**	35	15	2	0	52
**2: poor**	74	37	8	30	149
**3: somewhat poor**	0	0	0	4	4
**4: moderately well and well**	4	5	3	43	55
**5: somewhat excessive and excessive**	0	0	1	75	76
**Total**	113	57	14	152	336

^a^For all plots with potential tall shrubs present and a soil description available (results are similar for the full data set). Cell values are counts of plots in each cross-tabulated group

Flooding is associated with specific drainage and thaw depth classes (Tables 3 and 4). Alluvial deposition inhibits the formation of thick surface organic horizons and their associated shallow thaw (thaw class 1) and very poor drainage (drainage class 1; note the low cumulative binomial probability values for these drainage and thaw classes in Tables 3 and 4). Floodplains are one of the few places on the arctic landscape where a moist, loamy soil with gentle slope (drainage class 4) and relatively deep thaw (class 3 or 4) can occur (note the high probability values for these drainage and thaw classes in Tables 3 and 4), because in the study area soil development and plant succession on these soils produces thick surface organic horizons, poor drainage, and shallow permafrost.

**Table 3:** Correspondence of Soil Drainage Classes and Flooding^a^.

Soil Drainage Class	Count of Plots		Proportion of Flooded Plots	Probability^b^
	No Flooding	Flooding		
**1: very poor**	50	2	0.04	0.00
**2: poor**	111	38	0.26	0.53
**3: somewhat poor**	4	0	0.00	0.31
**4: moderately well and well**	30	25	0.45	1.00
**5: somewhat excessive and excessive**	55	21	0.28	0.71
**Total**	250	86	0.26	

^a^For all plots with potential tall shrubs present and a soil description available (results are similar for the full data set).

^b^Cumulative binomial probability of the observed count of plots with flooding ("successes") given the total number of plots in the soil drainage class ("trials") and a hypothesized probability of 0.26, the overall proportion of flooded plots.

**Table 4:** Correspondence of Soil Thaw Depth Classes and Flooding^a^.

Soil Thaw Depth Class	Count of Plots		Proportion of Flooded Plots	Probability^b^
	No Flooding	Flooding		
**1: < 40 cm**	97	16	0.14	0.00
**2: 40–60 cm**	39	18	0.32	0.88
**3: 60–80 cm**	8	6	0.43	0.96
**4: >80 cm**	106	46	0.30	0.92
**Total**	250	86	0.26	

^a^For all plots with potential tall shrubs present and a soil description available (results are similar for the full data set).

^b^Cumulative binomial probability of the observed count of plots with flooding ("successes") given the total number of plots in the soil thaw depth class class ("trials") and a hypothesized probability of 0.26, the overall proportion of flooded plots.

As will be discussed below in the Results, our wildfire-affected plots were too few to analyze the association between fire and other environmental variables.

The other environmental variables are weakly correlated, though thanks to the large sample size (n = 344) all pairs with r-squared of greater than 0.02 have significant probability values (P < 0.05; Table 5). In most cases less than 5% of the variance in the continuous variables pH, snow loss day, or July mean temperature can be explained by any one of the other variables (r-squared is less than 0.05). Exceptions include the correlations of soil pH with the categorical variables thaw depth, drainage, and flooding. Higher correlation in these cases results from the lower pH of wet, organic soils with shallow thaw and higher pH of soils where mineral bases are added by flood deposition. Flooded plots are also correlated with July mean temperature due to the generally lower elevations (and thus warmer temperatures) of river valleys. Nonetheless, 25% or less of the variance in pH, snow loss day, or mean July temperature is explained by thaw depth, soil drainage class, or flooding.

**Table 5:** Coefficients of Determination (R-squared) Matrix for the Environmental Variables^a^.

	Continuous Variables		Categorical Variables		
Continuous variables	Snow Loss Day	July Mean T,°C	Thaw Depth Class	Drainage Class	Flooding
**pH**	0.033*	0.025*	0.250*	0.159*	0.198*
**Snow Loss Day**		0.047*	0.017	0.042*	0.000
**July Mean T**			0.024*	0.005	0.109*

^**a**^For all plots with potential tall shrub species present (n = 344). For pairs of continuous variables these are linear regression R-squared values; for pairs of one continuous and one categorical variable they are the R-squared for a one-way analysis of variance. Asterisks (*) indicate F-test probability values of less than 0.05.

#### Analysis of shrub canopy volume and environmental factors

Plant abundance measures typically respond to a multiplicative combination of the predictor variables (environmental factors), and unsuitability of any one factor is sufficient to cause low abundance [33]. The resulting distribution of plant abundance along an environmental gradient has a “solid” distribution [34]: even at the point of optimum conditions along the environmental gradient, the plant will be absent or have low abundance in many samples due to unsuitability of other factors. Modeling of a species response to an environmental factor is challenging, as numerous sites provide little or no information about the gradient in question because they are unsuitable for some other reason.

Therefore, to understand the effect of an environmental factor we concentrate on samples where other factors are not strongly limiting, i.e. the highest values for the response variable (shrub canopy volume) at a particular point on the environmental factor gradient. A *single* observation of high canopy volume at some point on the environmental gradient (e.g. an alder canopy volume of 2 m at soil pH of 5) provides hard evidence that this species is able to form large canopies at pH 5. The absence of a species, or presence only with small canopy volume, in *all* plots in the sample across some range of values on the gradient (e.g. *Salix alaxensis* absent at pH 4 to 5) is weak evidence that this environmental condition is unsuitable for the species, because limitations in other factors may actually be the cause. We gain confidence that, for example, a soil pH of 4 to 5 is truly unsuitable for *S*. *alaxensis* only after investigating numerous locations in this pH range with a wide range in other environmental factors and failing to ever find a large *S*. *alaxensis* canopy.

Scatter diagrams of the response variable (shrub canopy volume) vs. the environmental variables are especially useful for discerning the shape of the species response surface, especially when sample sizes are not large [35]. Inspection of these scatterplots showed that the maximum shrub canopy volume had a single-humped distribution along the pH and snow loss date gradients. Thus quantile regression [41,42] was used to fit normal distribution curves to the highest shrub canopy volumes along these two gradients. I used the 0.95 quantile to approximate the form of the maximum envelope around the data. The “nlm” (non-linear minimization) function in the “stats” package of R statistical software [43] was used to fit the curves by successive approximation. In quantile regression, points above the curve are weighted by the quantile amount (here, 0.95) and points below are weighted by 1 minus the quantile (0.05), and the curve is fit by minimizing the sum of absolute deviations of points from the curve. While higher quantiles in principle are closer to the true maximum, they are progressively more influenced by fewer data points and thus can produce irregular results. Because some deliberate choice was involved in our sample locations, and plot locations were clustered in space, these quantile curves may not match the "true" 95^th^ percentiles of a probabilistic sample, but this was not the objective. The purpose of the regression was to discern the form of the maximum envelope around the data, and of the many methods tested, quantile regression produced curves with forms closest to my subjective concept of the maximum envelope.

The scatterplots of canopy volume vs. mean July temperature showed an abrupt rise in maximum volumes with rising temperature. Shrubs thickets composed of the species studied here are common throughout central and southern Alaska at localities warmer than the study area, and thus our data is not useful for locating the upper temperature limit of these species. To determine where shrub volume is most sensitive to rising temperatures, a sigmoid curve of the form *y = a/(1 + e* ^*b(c—x)*^ *)* was fit to the 95^th^ percentile values using quantile regression, where *y* is the canopy volume, *x* is July mean temperature, *a* is the amplitude of the rise in y, *b* is a constant that determines the rate of rise, and *c* is the temperature at the midpoint (steepest point) of the rising portion of the curve.

For the class environmental factors variables soil thaw depth, soil drainage, and flooding, the 95th percentile values for canopy volume were computed in each class.

As an additional test of the relationship between tall shrubs and July temperatures, I overlaid the tall shrub ecotypes in ARCN as mapped by classification of Landsat imagery ([24]; 30 m resolution) on the PRISM [19] July average temperature raster and computed the area occupied by tall shrub communities in each 1°C temperature class. "Ecotypes" here are vegetation types enhanced with some soil and landform information [24]. For comparison, I did the same for all forest ecotypes mapped in [24]. As an additional test of the relationship between tall shrubs and the end of snow season in tall shrub environments, I overlaid the tall shrub ecotypes from [24] on the map of end of snow season that was used above to obtain the plot end-of-snow-season values [36].

Error analysis of the ecotype map indicates that accuracy of mapping tall shrub ecotypes was quite good: 94% of tall shrub training pixels were mapped correctly as tall shrub [24]. The ecotype most often confused with tall shrub (2% of pixels) was Riverine Poplar Forest, which has ecological tolerances very similar to the closely associated riverine tall shrub type. An independent data set would probably produce lower classification accuracies.

#### Prediction of potential shrub expansion areas

I identified areas most likely to experience future shrub increase in tundra by overlay of rasters with the modeled mean July temperatures [25] and suitable soil conditions based on ecotypes mapped by [24]. The temperature raster was used to identify areas that currently have mean July temperatures between 0 and 2°C below the threshold for tall shrub thickets to form, as determined from the plot data and justified in the Discussion section below. These are the temperature zones likely to become suitable for tall shrubs with 2°C of warming. Ecotypes with suitable soil conditions within this temperature zone were identified as follows. The ecotype of each plot in the present study was assigned by the classification system used in [24], and the soil drainage, soil thaw depth, and soil pH were summarized by ecotype. The results were cross-checked against similar data provided in the ecotype descriptions in [24]. The analysis of shrub canopy volume vs. soil drainage, soil thaw depth, and soil pH from the present study were then used to identify the subset of ecotypes with the most suitable soil conditions for high-volume shrub canopies. These conditions are: weighted mean soil pH greater than 5.5, drainage class 4 or 5, or the potential for drainage class 4 or 5 to develop by permafrost thaw, and not one of the “barrens” ecotypes (unvegetated alpine areas lacking soil, highly disturbed active river gravel bars, marine beaches, or young lava flows). These limits are based on the plot data and are justified in detail in the Discussion. The ecotypes layer was also used to remove areas that currently have tall shrub or forest vegetation, since the goal was to locate areas of new shrub expansion on tundra.

Classification accuracies based on training pixels for most of the ecotypes that meet the above criteria are better than 80% [24], though an independent data set would probably produce less impressive accuracies. However, some of these classification errors do not in fact result in errors of predicted shrub-susceptible areas, because both the true and erroneous classes are susceptible (or both not susceptible) to tall-shrub expansion. For example, one of the largest misclassification errors was due to confusion of Alpine Dryas Dwarf Shrub and Upland Sedge-Dryas Meadow, both of which have soils susceptible to tall shrub expansion by the criteria listed above.

## Results

Twelve shrub species were observed to grow more than 1 m tall on at least one plot in the study area. These potential tall shrub species were recorded at 344 of the 471 plots and at 23 of the 24 sample nodes (Table 6). Potential tall shrub species were absent entirely from one node located on a barrier island of the Bering Sea (Fig 1). *Betula* sp. and *Salix pulchra* were the most frequent species. *Alnus viridis* and *Salix alaxensis* were the tallest and had the maximum observed canopy volumes. *Betula* sp. was the shortest of the study shrubs; it is dominantly the dwarf tundra form of *B*. *nana* in the study area.

**Table 6:** Summary Statistics of Common Potential Tall Shrub Species.

Species^a^	Count of Nodes^b^	Count of Plots	Median of Shrub Canopy^c^			Maximum Canopy Volume, m
			Cover, %	Height, m	Volume, m^d^	
***Alnus viridis***	9	36	22	1.67	0.40	2.6
***Betula nana + glandulosa***	23	247	8	0.14	0.01	0.3
***Salix alaxensis***	11	36	9.5	1.55	0.17	1.9
***Salix glauca***	13	48	3	0.44	0.01	0.3
***Salix pulchra***	20	121	5	0.30	0.01	1.2
***Salix richardsonii***	12	46	4	0.66	0.02	0.7
**All tall shrubs**	23	344	7.5	0.27	0.02	2.7

^a^Data for individual species are given only for the 7 most common species. Additional minor species include: *Salix arbusculoides* (4 plots), *S*. *bebbiana* (2 plots), *S*. *hastata* (16), *S*. *niphoclada* (9), *S*. *pseudomonticola* (4), and hybrid shrub-tree birches (*Betula glandulosa* or *B*. *nana* x *B*. *neoalaskana*, 3 plots)

^b^Nodes are sample localities with approximately 20 plots

^c^Median for the set of plots where the species was recorded

^d^Canopy volume in m^3^ per square meter of area, which reduces to meters

**Fig 9:**
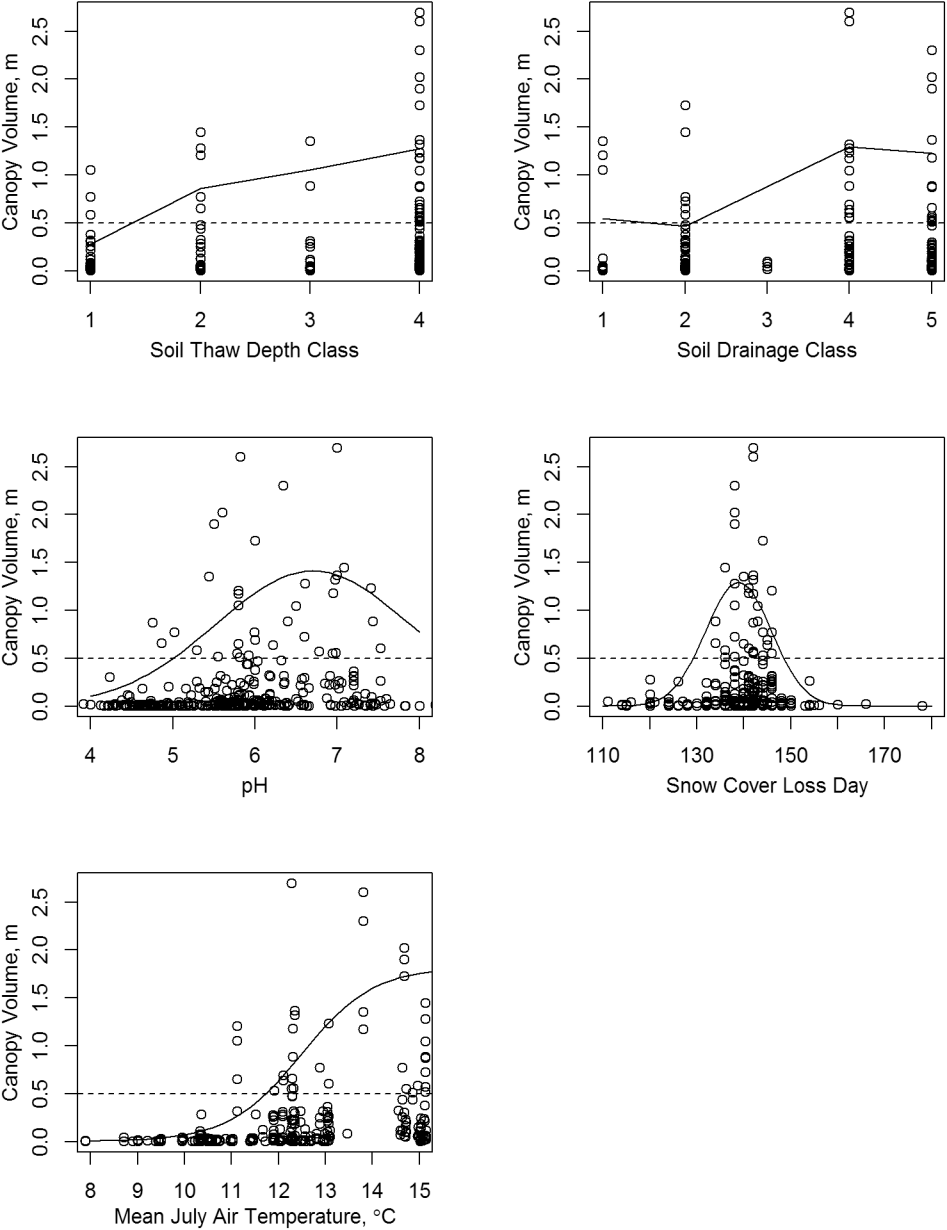
Scatter Plots of Shrub Canopy Volume vs. Environmental Factors for All Potential Tall Shrub Species together. The solid lines are curves fitted to the 95th quantile. The dashed lines mark a canopy volume of 0.5 m.

**Fig 10:**
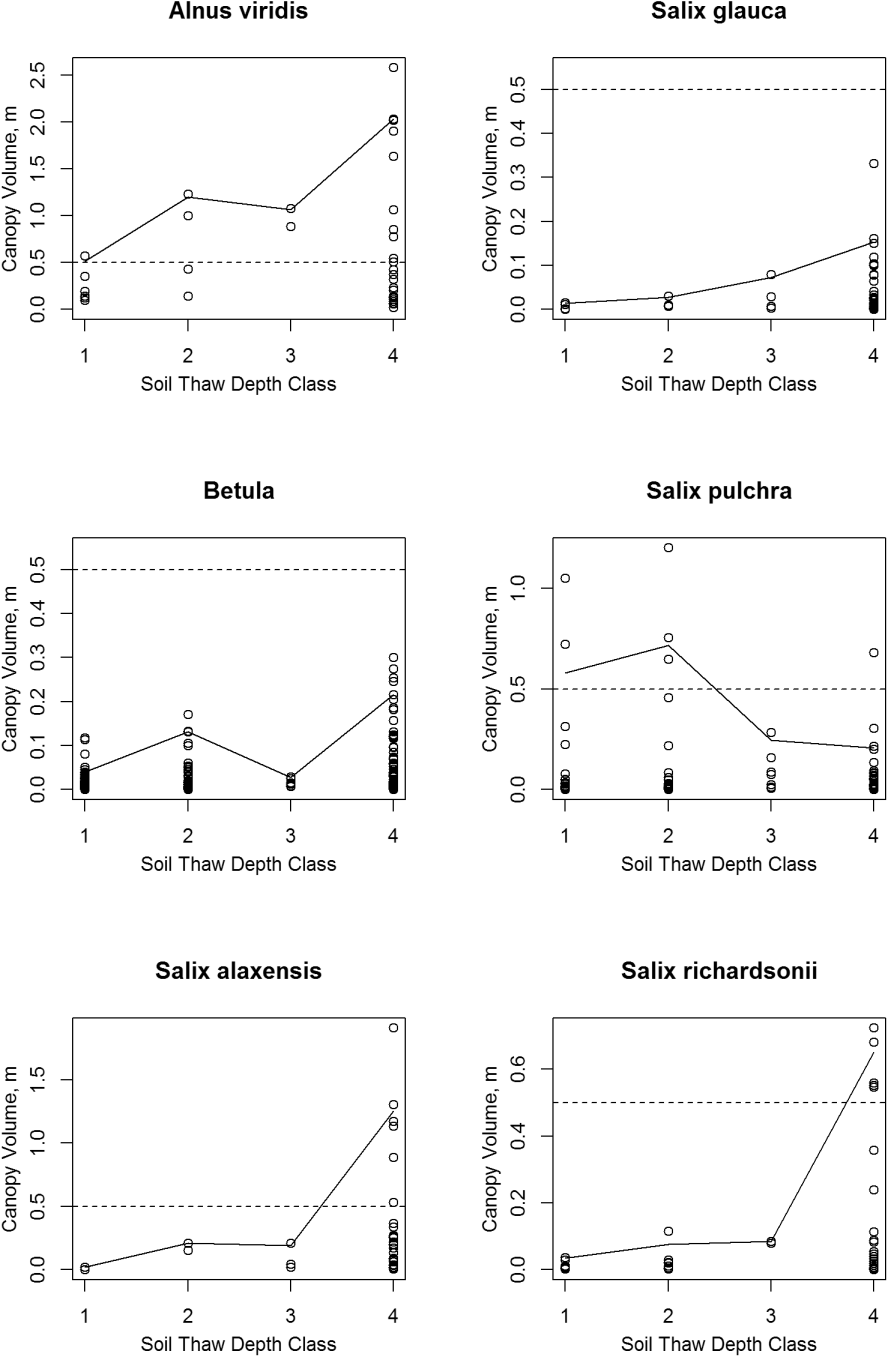
Scatter Plots of Shrub Canopy Volume vs. Soil Thaw Depth Class. The solid lines connect the 95^th^ percentile value in each class. Note that vertical scale varies between species; the dashed lines mark a canopy volume of 0.5 m.

**Fig 11:**
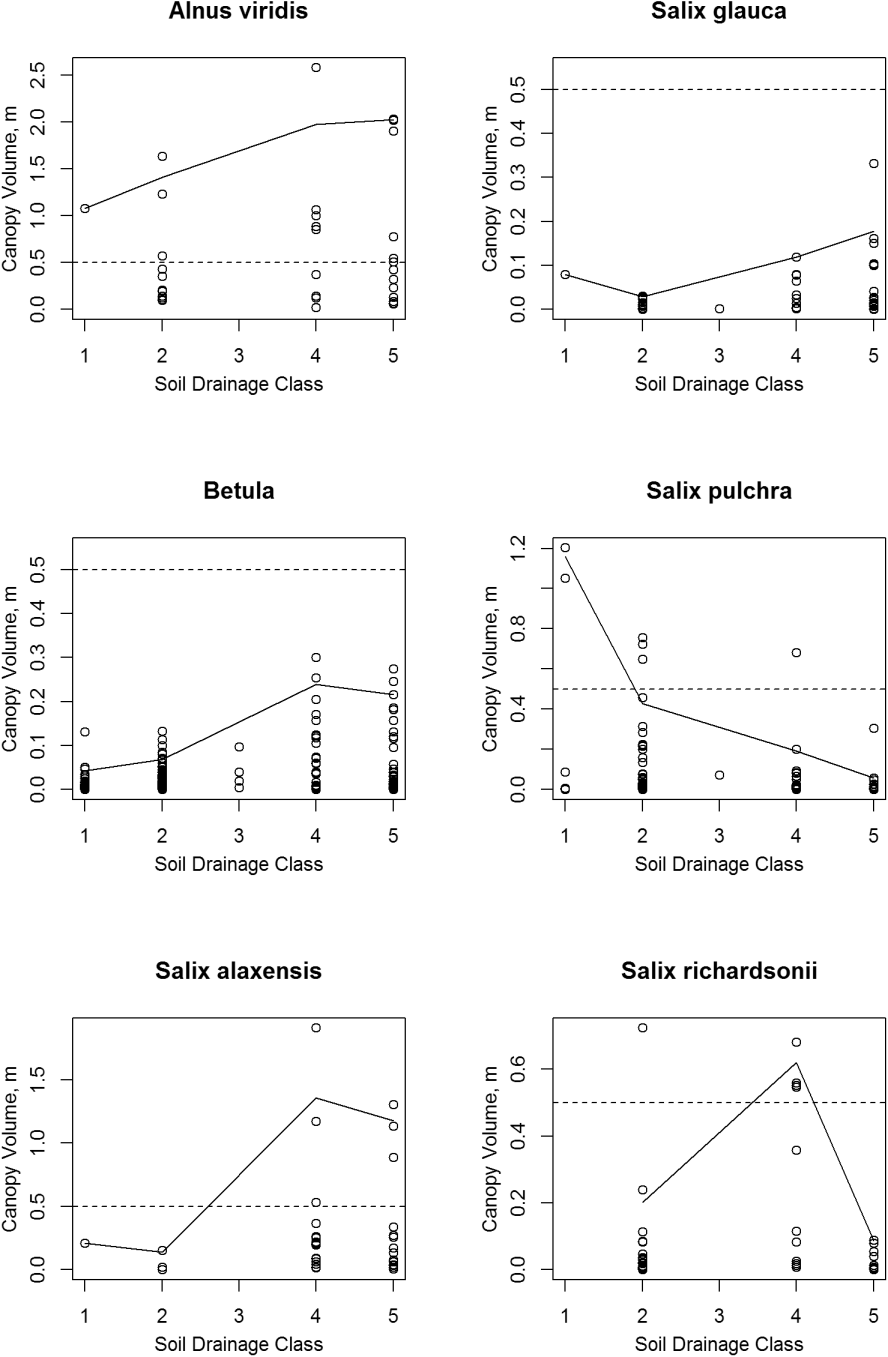
Scatter Plots of Shrub Canopy Volume vs. Soil Drainage Class. The solid lines connect the 95^th^ percentile value in each class. Note that vertical scale varies between species; the dashed lines mark a canopy volume of 0.5 m.

**Fig 12:**
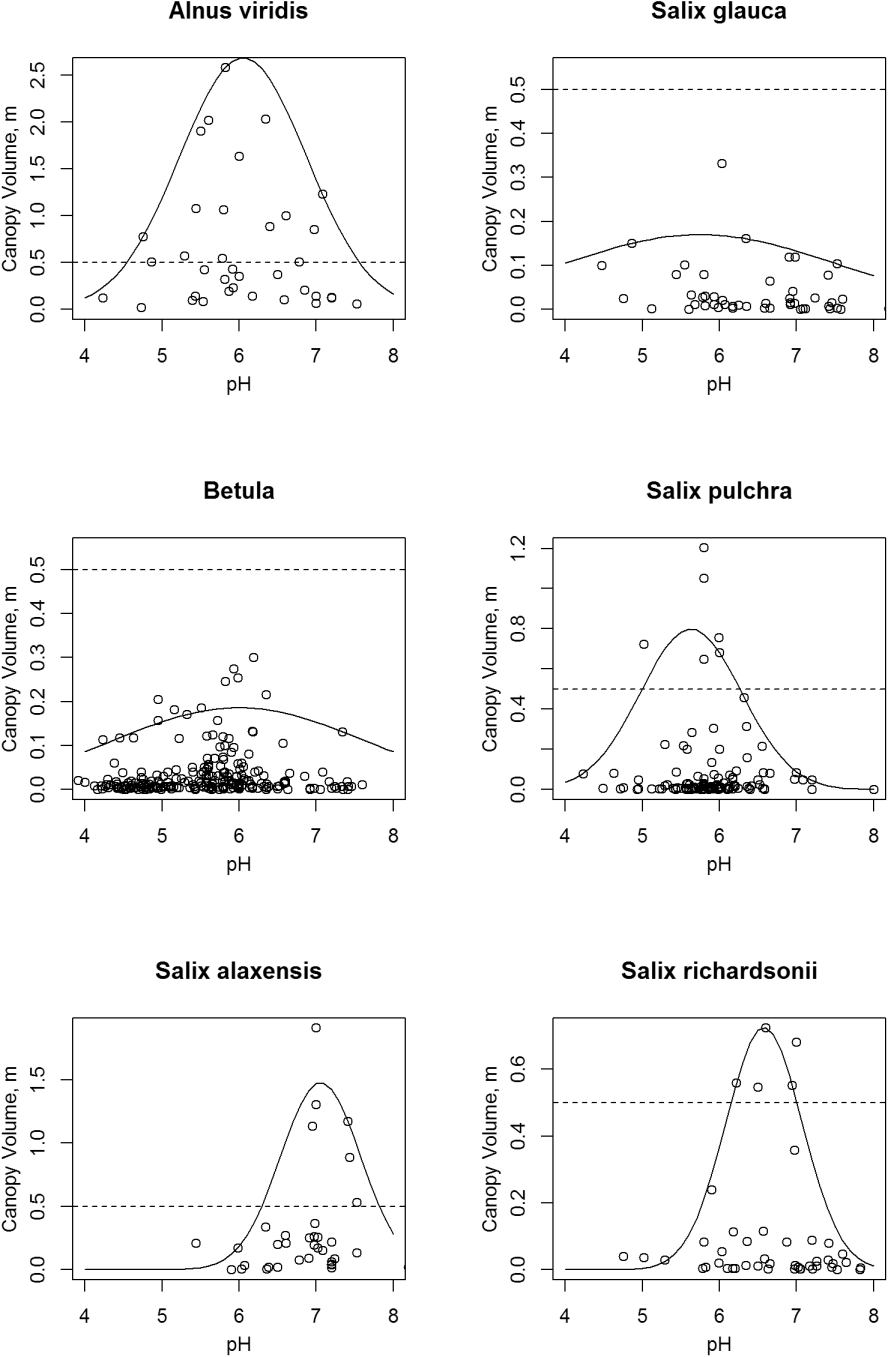
Scatter Plots of Shrub Canopy Volume vs. Soil pH. The solid lines are normal curves fitted to the 95th quantile. Note that vertical scale varies between species; the dashed lines mark a canopy volume of 0.5 m.

**Fig 13:**
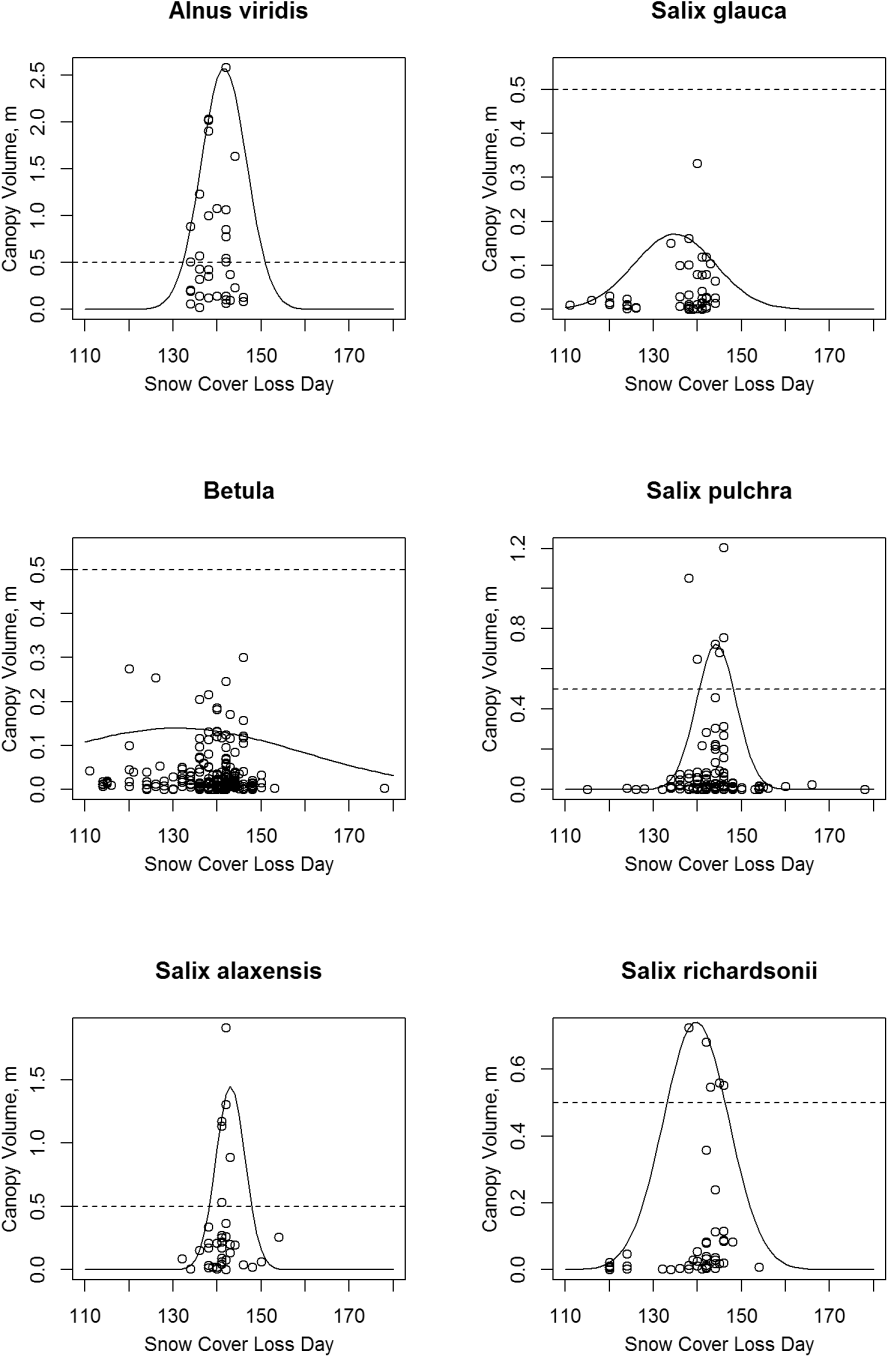
Scatter Plots of Shrub Canopy Volume vs. Date of Snow Cover Loss. The solid lines are normal curves fitted to the 95th quantile. Note that vertical scale varies between species; the dashed lines mark a canopy volume of 0.5 m.

**Fig 14:**
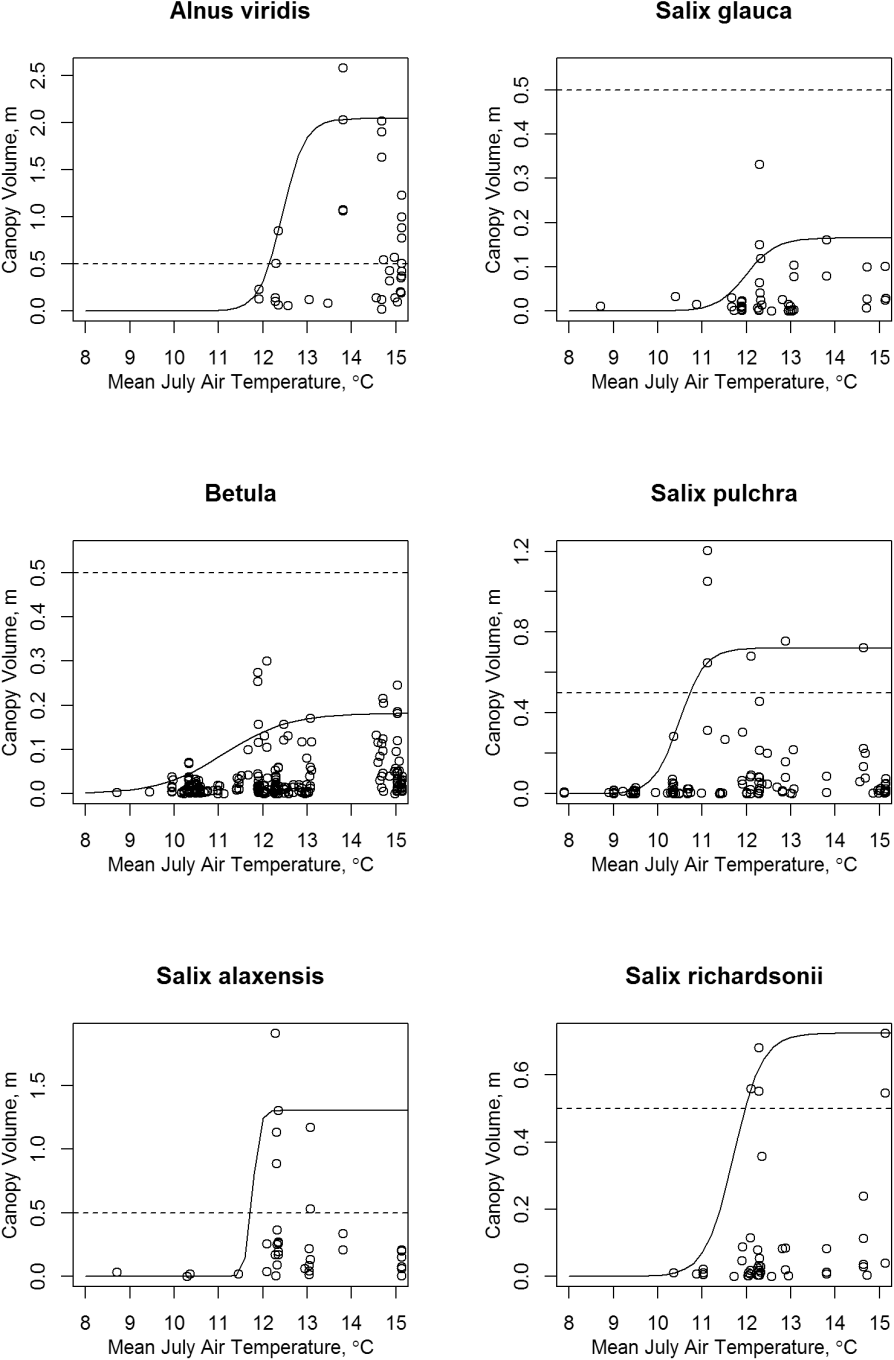
Scatter Plots of Shrub Canopy Volume vs. July Mean Air Temperature. The solid lines are sigmoid curves fitted to the 95th quantile. Note that vertical scale varies between species; the dashed lines mark a canopy volume of 0.5 m.

High shrub canopy volumes (defined here as canopy volume of over 0.5 m for all species aggregated) occurred on 36 plots at 10 different nodes. Recall that by the convention used here, a canopy volume of 0.5 m is equivalent to a 100% cover by a canopy 0.5 m tall, or 50% cover of a canopy 1 m tall. Environmental conditions on the plots with high canopy volumes spanned the full range of soil thaw depths classes (1 through 4) and soil drainage classes (1 through 5); they were on soils with mean rooting zone pH of 4.8 to 7.5, on sites with estimated July mean temperature of 11 to 15°C, and had snow cover loss dates of 134 (May 13) to 146 (May 25)(Fig 9). Plots of individual species vs. the environmental factors are presented in Figs 10–14 and discussed below.

### Soil Thaw Depth

The total shrub canopy volume was highest in soils with the greatest thaw depth (depth class 4: frozen soil deeper than 80 cm or frozen soil not present; Fig 9). All of the study shrub species except *Salix pulchra* had their greatest canopy volume in soil thaw class 4 (Fig 10). The shrub species varied greatly in their ability to grow canopies under colder soil conditions. Only alder (*Alnus viridis*) and *S*. *pulchra* attained canopy volumes of 0.5 m or greater in soil thaw classes 1 and 2 (soils that thawed to less than 60 cm). *Betula* sp. were quite common on shallow-thawing soils and reached roughly half as much volume in the shallow thaw classes 1 and 2 as they did in class 4, but owing to their overall smaller stature, their maximum volumes on cold soils were much smaller than alder or *S*. *pulchra*.

### Soil Drainage

The total shrub canopy and four of the six individual shrub taxa had their highest canopy volumes on drier soils of drainage classes 4 and 5 (Figs 9 and 11). *Salix pulchra* was again an exception; its highest canopy volumes were on the wettest soils of class 1 (very poorly drained), but it was actually much more common on soils of class 2 (poorly drained). *Salix richardsonii* had one high canopy volume measurement on drainage class 2, but appeared to peak in the mesic soils of drainage class 4 and decline in droughty class 5 soils. Alder had relatively high canopy volumes of over 0.5 m across the full range of wetness classes.

### Soil pH

The largest total shrub canopy volumes occurred in the range of soil pH 6 and 7 (Fig 9). Four species had distinct optimal pH values, as taken from the fitted curves (Fig 12): *Salix alaxensis* (7.1), *S*. *richardsonii* (6.6), *A*. *viridis* (6.1), and *S*. *pulchra* (5.6). Alder and *S*. *pulchra* attained high canopy volumes (over 0.5 m) on soils down to a pH of about 5, while *S*. *alaxensis* and *S*. *richardsonii* had high volumes only above pH 6. The *Betula* sp. were by far the most common shrub on the very acid soils with pH below 5, but their canopy volumes under these conditions were quite small, less than 0.2 m.

### Snow Cover Loss Date

Shrub thickets with volumes of 0.5 m or more (all species combined and the individual species) had normal snow cover loss dates that were restricted between days 134 (May 14) and 146 (May 26; Figs 9 and 13). The *Betula* sp. were the most common taxa on sites with snow loss prior to day 134 (May 14), though as a result of this species’ small stature, canopy volumes there were less than 0.3 m.

According to GIS overlay of tall shrub communities as mapped with Landsat imagery on the map of normal snow cover loss dates [24,36], 80% of the tall shrub area had snow-free dates between 134 and 153 (14 May to 2 June).

### Mean July Air Temperature

High canopy volumes were observed only at sites with mean July temperature of 11°C or greater (Fig 9). The midpoint of the rising portion of the fitted sigmoid curves marks the steepest slope and thus is a good estimate of the threshold temperature separating low and high canopy volumes (Fig 14). These threshold temperatures were, in increasing order: *Salix pulchra* 10.5°C, *Betula* sp. 11.1°C, *Salix richardsonii* 11.7°C. *Salix alaxensis* 11.8°C, *Salix glauca* 12.0°C, and *Alnus viridis* 12.4°C. Only *Salix pulchra* reached high canopy volumes (>0.5 m) below 12°C. Alder reached its maximum volumes at temperatures of 14° to 15°C.

**Fig 15:**
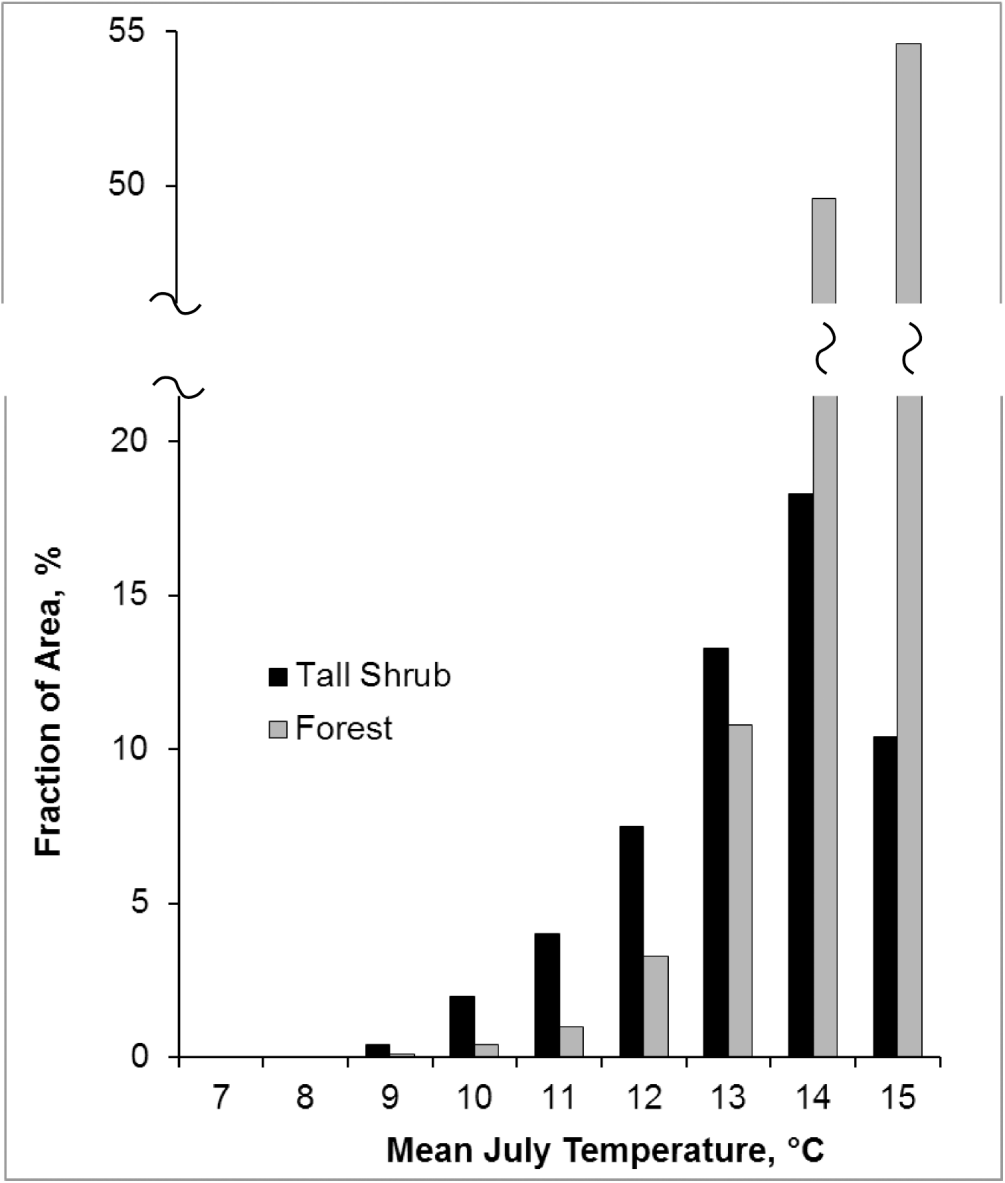
Proportion of ARCN Land Area with Tall Shrub or Forest Vegetation, by July Mean Temperature Class. Temperatures are rounded to the nearest °C to form classes. For example, of the land area in the 12°C class (temperature 11.5°C to 12.5°C [25]), 3.3% was in forest and 7.5% in tall shrub vegetation [24].

Overlay of the tall shrub ecotypes in ARCN as mapped by classification of Landsat imagery on the PRISM July average temperature raster [24,25] showed that the proportion of the landscape with tall shrub vegetation was minimal in areas with July average temperature below 10°C and increases with increasing July temperature until overtaken by forest above 13°C (Fig 15).

### Disturbance

Just 14 plots had been affected by wildfire. Eight were tussock tundra plots that had burned in 2010 before sampling in 2011, and only small amounts of re-sprouting shrubs were present (canopy volumes less than 0.035 m). The remaining six burned plots were in a black spruce (*Picea mariana*) and aspen (*Populus tremuloides*) woodland that burned in 1991 and were sampled in 2013. These plots had open stands of small trees with shrubs present, mainly *Betula sp*. Shrub canopy volumes were modest, 0.05 to 0.23 m.

About one fourth of the 344 plots with potential tall shrub species present were affected by floods. The very highest canopy volume plot (2.7 m) was on a floodplain site, and 22 of the 36 high-volume canopies (> 0.5 m) were flood-affected. Most of the willows attained markedly higher canopy volumes on flooded plots than non-flooded plots (Table 7). The largest alder canopies were on un-flooded plots, though some high-volume alder canopies were also present on floodplains.

**Table 7:** Canopy volume of potential tall shrubs in relation to flooding.

Species	95^th^ percentile canopy volume		Count of Plots	
	No Flooding	Flooding	No Flooding	Flooding
***Alnus viridis***	2.03	1.07	21	15
***Betula***	0.13	0.13	205	42
***Salix alaxensis***	0.29	1.26	8	28
***Salix glauca***	0.16	0.12	30	18
***Salix pulchra***	0.28	0.69	87	35
***Salix richardsonii***	0.08	0.64	18	28
**All Potential Tall Shrubs**	0.52	1.271	258	86

### Future Shrub Expansion

**Fig 16:**
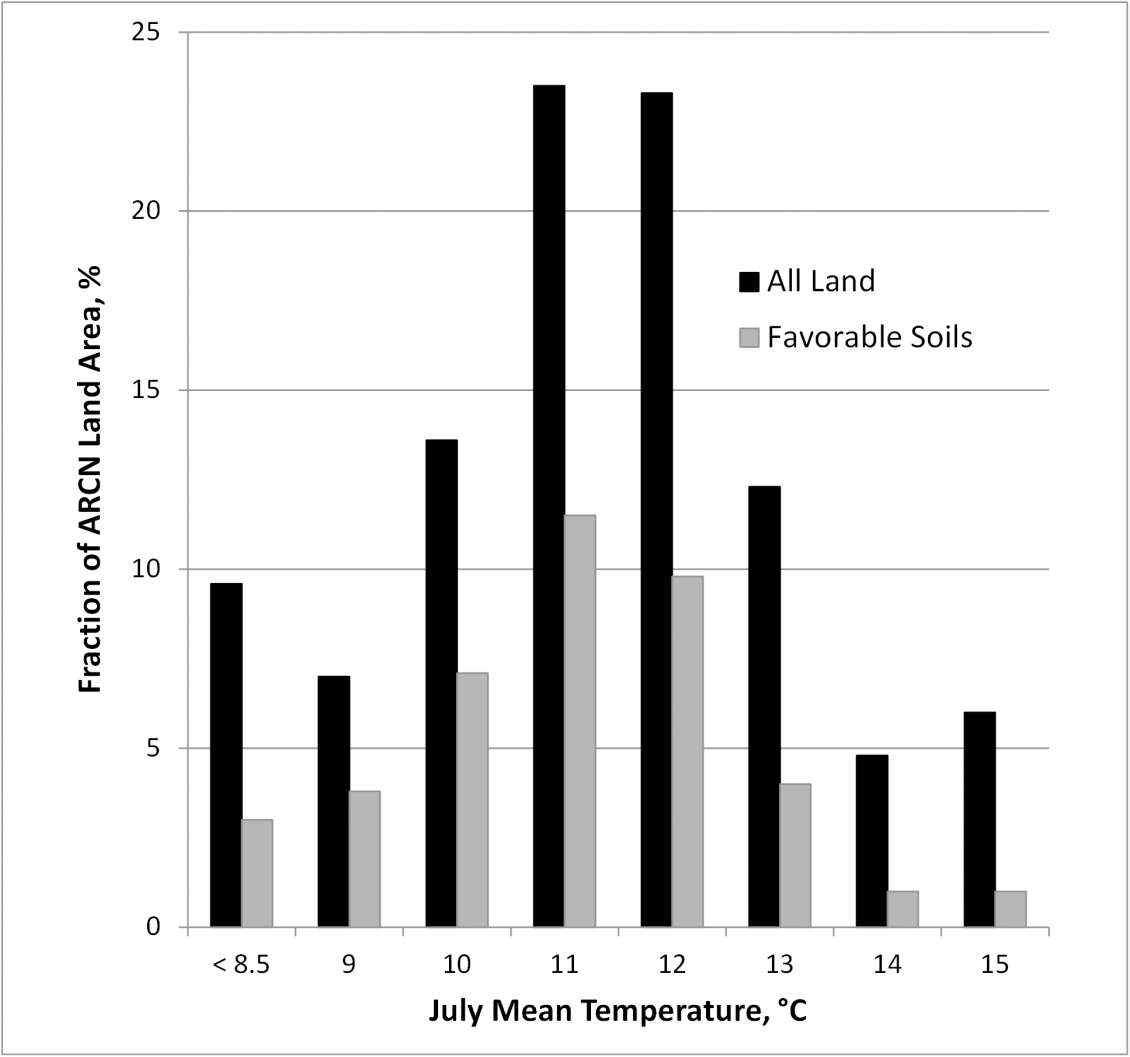
Area of ARCN with Various July Mean Temperatures and Soils Favorable to Shrub Expansion. July mean temperatures are from the PRISM Climate Group [25] modeled raster, rounded to the nearest °C. “Favorable soils” are the area within each July temperature class with soils favorable to the growth of potential tall shrub species, as mapped by Jorgenson et al. [24] and discussed in the text. Areas currently with tall shrub or forest vegetation were excluded.

Currently about 23% of ARCN has estimated mean July temperatures of 11.5°C to 12.5°C (i.e. just below the threshold for alder and near the threshold for *S*. *richardsonii*, *glauca*, and *alaxensis*). Another 24% of ARCN area has estimated mean July temperatures of 10.5°C to 11.5°C (Fig 16). Thus nearly half of ARCN area has mean July temperatures of 0 to 2°C below the threshold for multiple tall shrub species. Soils favorable for shrub expansion occupy just under half of the land area within these two temperatures zones (Fig 16).

**Fig 17:**
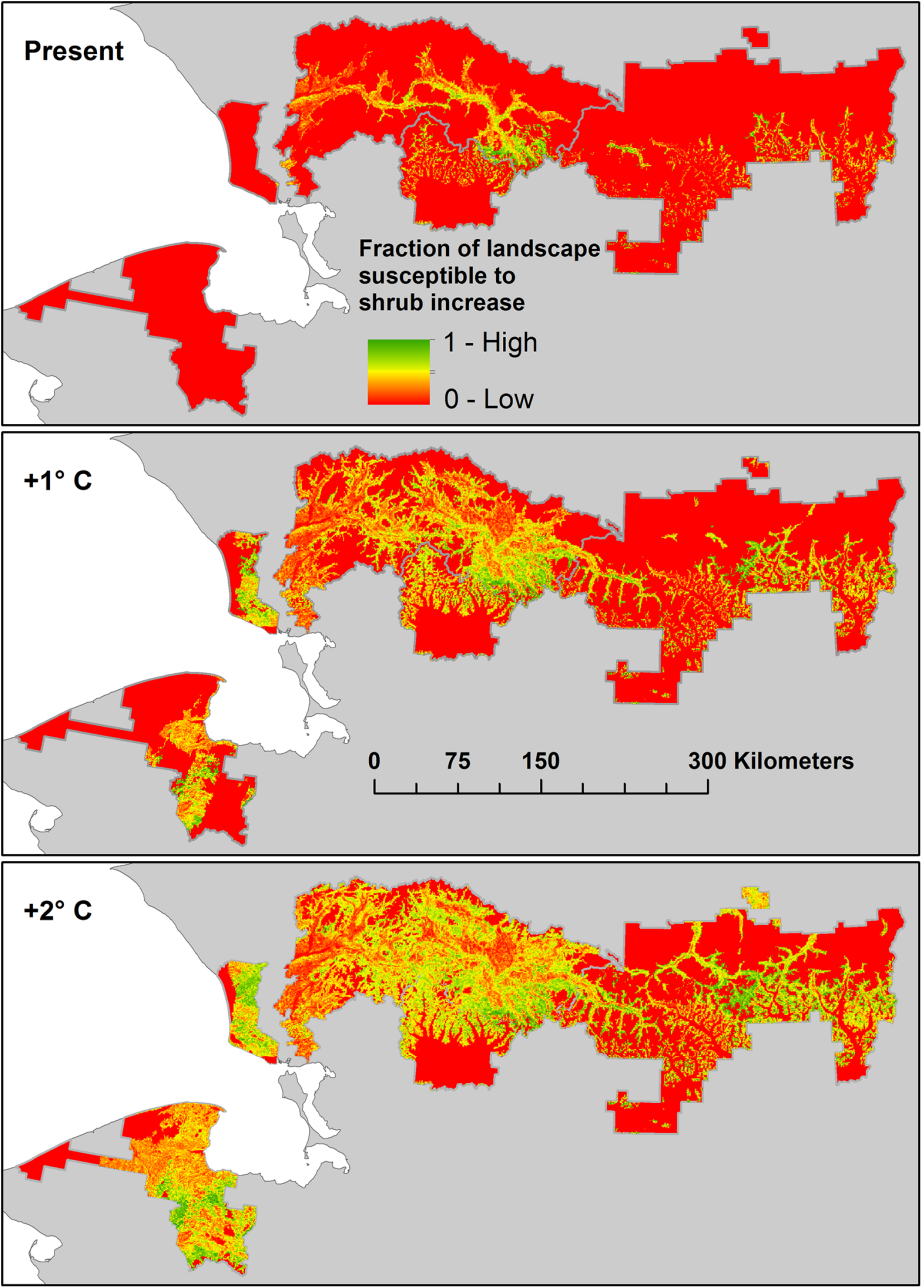
Susceptibility Map for Shrub Expansion on Tundra in ARCN. Susceptibility refers to the proportion of the landscape with soils favorable to the formation of high-volume shrub canopies, and ranges from 1.0 (“High”, green) to 0 (“Low”, red). The maps portray the proportion of soils with high susceptibility in areas of present-day mean July temperature of 12.5 to 13.5°C (top), 11.5 to 13.5°C (middle), and 10.5 to 13.5°C (bottom). These simulate the areas favorable to shrub expansion at the present time and with one and two degrees of additional summer warming ("present", "+1°C ", and "+2°C ").

Susceptibility maps for tall shrub expansion in ARCN (Fig 17) show where the greatest opportunities for shrub expansion will be with varying degrees of future warming. Areas with favorable soils and present summer temperatures of 12.5–13.5°C (Fig 17, top) are near the present-day temperature threshold for high-volume shrub canopies and thus should currently be experiencing shrub expansion due to recent warming. The Noatak River valley and areas near treeline in the mountain valleys on the south slope of the Brooks Range are the main areas affected. Suitable soils in areas with July temperatures of 11.5–13.5°C (Fig 17, middle) and 10.5–13.5°C (Fig 17, bottom) add areas that should be favorable for shrub expansion with one and two degrees of additional summer warming, respectively. These cover the inland portions of BELA and CAKR, much of the Noatak River basin, and the mountain valleys of GAAR.

## Discussion

Large shrub canopy volumes are constrained in ARCN by multiple environmental factors, and hence they are relatively rare. While temperature is clearly an important limiting factor on tall shrub growth, other soil and site factors are important also. Limitations imposed by other environmental factors restrict shrub communities to certain sites within the zone of adequate summer warmth, and would continue to do so under a warming climate. These limitations include environmental factors quantified by our other site variables (soil thaw depth, soil drainage, soil pH, snow cover, and disturbance), plus plant competition, lack of propagules, and herbivory. A discussion of each of these factors follows.

### Mean July Air Temperature

The threshold values for high shrub canopy volumes from the present study of 10.5°C (*Salix pulchra*) to 12.4°C (*Alnus viridis*) agree with results of my analysis of vegetation change in ARCN between 1980 and 2010 based on aerial photograph interpretation of 206 systematically located plots [4]. In that study, 15 photo-interpreted plots experienced shrub expansion on tundra, and all had July average temperatures between 11.3°C and 12.9°C, except one at 14.1°C (according to the same PRISM [25] temperature maps used here). Alder seed viability is negligible when mean summer temperatures are below 10°C (equivalent to about 12°C mean July temperature) and increases markedly as temperature rises [44].

Thus multiple lines of evidence suggest that temperature thresholds for the various tall shrub species in ARCN are between 10.5 and 12.5°C. Summer temperatures in this part of the arctic are expected to rise an additional 1 to 2°C by the middle of this century, based on modeling using the moderate RCP4.5 emissions scenario [45]. Such a rise would be a bit faster but still comparable to the historical rate of rise in July temperatures of about 1.7°C/century at Kotzebue and Bettles over the past 65 to 80 years (Fig 6). A uniform rise of 2°C July temperature across ARCN would put 70% of the area into the optimal temperature range for all of the study shrub species (over 12.5°C), compared to just 23% at the present. In other words, the area with optimal temperatures for tall shrub thickets in ARCN could approximately triple due to warming during the next century.

It is possible that shrubs in some places have not had time to reach the full canopy volumes possible under the present climate, as a result of 20th-century climate warming and the lag time required for shrubs to disperse, become established, and grow. Thus I may have slightly overestimated the equilibrium temperature requirements of shrub species because canopies have not fully adjusted to newly warmed temperatures. These errors due to disequilibrium conditions and also any possible systematic bias in the PRISM temperature grids should have minor effects on our interpretations, which are based on assessment of relative change (e.g. a future increase of 2°C) rather than exact knowledge of current conditions.

### Soil pH, soil thaw depth, soil drainage, and disturbance

Soil acidity limits the extent of tall shrubs in ARCN. Soil pH is most limiting for our largest common willow, *S*. *alaxensis*, which requires near-neutral soils. Near-neutral soil pH values are restricted in the study area to uplands underlain by carbonate bedrock, and to floodplains. Most other ecotypes in ARCN have soil pH around 6, and thus have pH suitable for growth of the other tall shrub species. However, significant areas have soil pH well below 6, and tall shrub growth is limited by soil acidity. The pH of the very widespread ecotype “Upland Dwarf Birch-Tussock Shrub” (pH 5.1±0.6; 20% of ARCN) is near the lower limits of alder and *S*. *pulchra*. Thus acidity, along with shallow thaw (thaw class 1) and poor drainage (drainage classes 1 and 2) limit tall shrub growth on the very widespread tussock tundra ecotype.

Since the largest shrub canopy volumes occur on relatively deep-thawing and dry sites, we would expect warming-related shrub expansion to occur there. Tape et al. [46] found alder expansion in arctic Alaska correlated with higher soil temperatures, deeper thaw, and lower soil moisture. The most common ecotype to convert to tall shrub in ARCN during the period 1980–2010 was “Upland Birch-Ericaceous-Willow Low Shrub” [11], which is a deep-thawing upland type that usually has good drainage [24].

The high canopy volumes of most species on the very droughty soils of drainage class 5 suggest that soil moisture is not strongly limiting for tall shrubs in the study area, except for *Salix pulchra* and *S*. *richardsonii*. Thus shrub expansion in general should not be constrained by warming-induced drought, as has been observed elsewhere for alpine vegetation [47,48].

Flooding is an important process that helps maintain high soil pH, deep thaw, and good drainage in the study area (Tables 3–5). The association of flooding with these favorable soil conditions for shrub growth, and the opportunities for shrub establishment on bare alluvium on some floodplain sites, are responsible for the observed high shrub canopy volumes on flooded sites (Table 7).

Fire is known to enhance shrub growth in the tundra [9,49,50], and this effect can persist for over a century after the fire [50]. Fires remove acid surface organic matter [51], which increases soil thaw depth and improves soil drainage [52]; these processes together allow plant roots to exploit the less acid subsurface mineral soil. (Fires also increase soil pH through direct release of bases from burned organic matter [53], but this effect is both transient and shallow.) Wildfire is relatively rare in the tundra portion of ARCN [15], and hence a small proportion of our plots have burned during the time of historical (post-1940) fire records.Future warming, or warming coupled with increased fire frequency, is likely to increase thaw depths, improve drainage, and raise the pH of soil available to rooting. The possible magnitude of this effect is difficult to predict, however. Fires alone can result in warming of the mean annual temperature of soils on permafrost by a few degrees [52], i.e. an amount comparable to that expected with climate change. Studies of post-fire thaw of permafrost in ARCN [35,49] show that poorly drained, level to gentle slopes (less than 10%), and concave lower slopes with thick organic surface horizons, are resistant to change in drainage conditions with warming. The most change-prone areas are moderate slopes (more than 10%) with planar to convex shape and fine-grained soils that are currently in drainage class 2 (poorly drained) and thaw class 1 or 2 (<40 cm and 40–60 cm). Warming or wildfire could shift these sites into thaw class 3 or 4 (>60 cm) and improve drainage to class 3 or 4 (somewhat poorly to well drained).

Areas of ARCN that are most likely to experience a shrub increase as a result of warming- or fire-induced deeper thaw and improved drainage can be estimated from the ecotypes [24] that have characteristics of thaw-susceptible areas as outlined above. The ecotypes most susceptible to conversion to tall shrub thickets by thaw of permafrost are probably “Lowland Birch-Ericaceous-Willow Low Shrub” (3% of ARCN), “Lowland Sedge-Dryas Meadow" (1%) and “Upland Sedge-Dryas Meadow" (6%). Much of the very widespread “Upland Dwarf Birch-Tussock Shrub” (20% of ARCN) should be resistant to thaw as a result of low slopes and thick organic surface layers. However, the more steeply sloping and convex areas in this ecotype could be susceptible to thawing and drying and possible shrub expansion. Because “Upland Dwarf Birch-Tussock Shrub” is currently not good tall shrub habitat and most would probably remain so with a degree or two of warming, it was not included in the list of ecotypes that might be colonized by tall shrubs used to produce Fig 17.

If mean annual temperatures in ARCN warm more than about 3°C (the amount projected by mid-century [45]), permafrost in large areas of lowland areas of ARCN would start to degrade [54], including the relatively thaw-resistant lowland organic ecotypes like " Upland Dwarf Birch-Tussock Shrub”. (Permafrost stability depends on mean annual temperatures, which are expected to change more than summer temperatures [45].) Extensive permafrost degradation would lead to widespread soil disturbance by subsidence and mass movement [55]. Shrub expansion would likely exceed what is portrayed in Fig 17, especially if wildfires also become more prevalent [51]. Mass movements triggered by permafrost thaw (thaw slumps and landslides) expose subsoil suitable for shrub colonization [56,57]. For this to become a significant factor in shrub expansion, the area covered by these features would have to increase significantly beyond its current extent of just a few hundred hectares across all of ARCN [58]. Cryoturbation (frost churning of soil), which would be a likely outcome of permafrost thaw, can also bring higher-pH mineral soil to the surface [59] and produce bare-soil sites favorable for shrub colonization [60]. This process would probably take decades to centuries to be effective over large areas.

### Snow cover

The range normal spring snow-off dates for tall shrub communities is quite narrow, ranging across just 12 days in May (days 134–146) by our plot data and about 19 days by GIS analysis (days 134–153). About 10% of ARCN area is typically snow-free on day 132 and 10% is still snow-covered on day 155 [36]. Locations in ARCN with very early snow cover loss are places where high winds in the winter remove snow, while late snow cover loss locations represent deep snowdrifts [37]. Thus about 80% of ARCN appears to have suitable snow conditions for tall shrubs: neither too wind-scoured nor too late to melt out in the spring.

*B*. *nana* is better adapted to windy conditions than the other shrubs discussed here, as shown by its ability to produce canopy volumes near the maximum for the species (0.3 m) in places with snow cover loss as early as about day 120 (April 30). *B*. *nana*’s tough waxy leaves and lower growth form are good adaptations to windy conditions. In very windy situations *B*. *nana* can take on a creeping growth form, similar to the arctic's many prostrate dwarf shrubs.

The multiple and possibly conflicting results of changes in the wind and snow regimes introduce uncertainty into the effect of climate change on the wind and snow cover factor in shrub growth. Warmer growing seasons would allow shrubs to grow faster and overcome the effects of winter scour or persistent snow. A decrease in winter wind could reduce both wind scoured areas and the adjacent snow beds, but the effect of climate change on winter windiness is unknown. The anticipated increase in winter precipitation in the arctic [45] would produce more and larger snow beds that inhibit shrub growth, but it could also protect shrubs in lightly wind-scoured areas. Earlier spring thaws could allow shrubs to expand into previous snow bed areas. Shrubs themselves influence snow depths by capturing snow, with potential positive feedbacks of warming soils and more protection from wind [8].

### Dispersal, competition, and herbivory

The broadly defined ranges of all of the potential tall shrub species considered here extend across all of ARCN [61,62], though local availability of propagules differs among them. Alder is uncommon in BELA and CAKR ([24,62], and the present study). *Betula* sp. and the willows, especially *S*. *pulchra*, are common throughout the study area (Table 6). The time required for dispersal, establishment, and growth obviously will delay plant community response to warming everywhere, but the effects should be most apparent for alder in BELA and CAKR.

Competition from trees has minor effects in our data set. Just 15 of the 471 plots in our data set had tree species present at over 25% cover, and 6 of those 15 plots nonetheless also had large (> 0.5 m) shrub volumes. (Tree species here include *Betula neoalaskana*, *Picea glauca*, *Picea mariana*, *Populus balsamifera*, and *Populus tremuloides*.) Competition from trees is probably responsible for the decline in shrub area in the warmest part of ARCN, where mean July temperature is 15°C (Fig 15), but this temperature zone occupies only about 6% of ARCN. While competition from a tree overstory occurs only locally in our study area, dense growth of low shrubs, herbs, and non-vascular plants undoubtedly inhibits establishment of potential tall shrubs species on tundra, even when soil conditions are suitable. The same disturbance processes discussed above that improve soil pH and drainage (fire, flooding, and ground mass movements due to permafrost thaw) also create opportunities for shrub establishment by removing competitors and exposing bare soil. Thus shrub expansion in densely vegetated lowland areas should occur most rapidly on floodplains and after fires.

Vertebrate herbivores with potential to limit shrub expansion include moose (*Alces alces*), caribou (*Rangifer tarandus*), muskox (*Ovibos moschatus*), beaver (*Castor canadensis*) snowshoe hare (*Lepus americanus*), Alaskan hare (*Lepus othus*) and ptarmigan (*Lagopus lagopus* and *L*. *muta*). All of these species prefer willows over alder or birch shrubs [63]. Birch shrubs in our study area are highly defended against browsing herbivores [64]. Multiple insect herbivores also affect all of the shrub species in ARCN [65]. Moose browsing effects on willows were observed at over half of the nodes in the study area. Hares and moose have increased in recent decades in tundra-forest transitional areas of northern Alaska [13]. Analysis of the potential effect of browsing is beyond the scope of the current study, but it is worth noting that the browsers’ preference for willow over alder or birch could tilt the competitive balance in favor of the latter two species.

### Future Shrub Expansion

The area of soils favorable for shrub expansion occupy just under half of the land area in the parts of ARCN expected to be favorable for tall shrub communities with about 2°C of warming (the current 10.5 to 13.5°C temperatures zone, 59% of ARCN area; Figs 16 and 17). The favorable soils in this temperature zone represent about 25% of the total area of ARCN. Most of the ecotypes involved already have a shrub component, and thus future shrub expansion would be by infilling of canopies, canopies becoming taller, and replacement of smaller-statured shrubs by taller species.

A state-and-transition model was used by Jorgenson et al. [21] to quantify the area expected to transition between ecotypes during the next century, by extrapolation of recent rates of change into the future. Their study area largely coincides with the present one. That study concluded that the area covered by tundra low shrub communities would decrease by 2 to 8% under various scenarios, mainly due to their replacement by tall shrubs, but the net gain in tall shrub communities would be less than 2% as a result of simultaneous replacement of the tall shrub communities by forest.

The apparently smaller change predicted by Jorgenson et al. [21] can be reconciled with the present study by noting that the 25% of ARCN area susceptible to shrub expansion represents the maximum potential area available, and the actual future shrub extent will be limited within this area by unquantified environmental limitations (e.g., wind), and by limitations to the rate of dispersal, establishment, and growth. Also, only a fraction of the area with shrub increase would undergo a physiognomic class change, e.g. from dwarf shrub tundra to tall shrub tundra, and thus be registered as a "change" by the state-and-transition model. Finally, the present study does not account for replacement of tall shrub communities by forest.

The vegetation change scenarios suggested by the present study appears less drastic than those implied by global-scale models [19,20], which predict biome changes over half of the arctic and northward biome shifts of hundreds of kilometers. The disagreement is due mainly to the finer spatial scale of the present study, which allows significant areas of the landscape to resist physiognomic change as a result of soil limitations.

The present distribution of tall shrub and forest communities in ARCN shows that tundra tall shrub communities occupy a rather narrow band on the temperature gradient just below forest (Fig 15). The relative area of tall shrub communities in the future could increase with warming because trees should be limited more by dispersal than shrubs. Only the tree *Populus balsamifera* is currently widespread in tundra areas of NOAT and hence well positioned for expansion with warming, but it is soil-limited to well-drained, non-acid sites (mainly floodplains) like *Salix alaxensis*. White spruce (*Picea glauca*) and Paper birch (*Betula neoalaskana*) have wider adaptability (similar to alder [66]) but these species currently only occur in the southern and lower-altitude parts of ARCN and not at all in BELA. The tree with the greatest potential to colonize ARCN's extensive tussock tundra with warming is black spruce (*Picea mariana*), well known for its tolerance of wet and acid soils [66,67]; but black spruce is currently only present in lowlands of the Kobuk River valley in areas with mean July temperature above 13°C. Substantial warming and migration over mountain barriers would be required to convert the extensive lowlands of NOAT, CAKR, and BELA to black spruce forest.

The different shrub species should react differently to changing environmental conditions. Alder requires more summer warmth than the other species analyzed here, but it thrives over a wide range of soil conditions. Thus warming should open up many new sites for colonization by alder. Since most of ARCN is already warm enough for *Salix pulchra* thickets, warming will provide fewer new opportunities for it to expand. Alder and *S*. *pulchra* overlap in their ability to grow on cold, wet, organic soils of moderately low pH, and climatic warming could lead to some replacement of *S*. *pulchra* by alder on these sites.

The narrow niche requirements of *S*. *alaxensis* (well drained soils with neutral pH) suggest that its expansion with warming will be limited to floodplains and upland areas with carbonate bedrock ([24] and this study). *S*. *glauca* and *S*. *richardsonii* are more tolerant of wetness (especially *S*. *richardsonii*) and acidity (especially *S*. *glauca*) than *S*. *alaxensis*, but alder is as well adapted to both wetness and acidity as any of these three willows, besides being less attractive to most herbivores.

The *Betula* sp. are already ubiquitous and well adapted to acidity, wetness, and cold. Increases in canopy volume by infilling and height increase of *Betula* sp. with warming are likely. Below 12°C mean July temperature *Betula* canopy volumes were nearly always less than 0.05 m, while above 12°C we found canopy volumes of 0.1 to 0.3 m. Though they are not tall shrub thickets, these latter volumes present significantly more competition to herbaceous plants and lichens than canopies below 0.05 m. Formation of tall *Betula* thickets would probably require gene flow from the south, since most occurrences of *Betula* shrubs over 1 m tall in the study area are by the southerly *B*. *glandulosa* species, and the more prevalent tundra species *B*. *nana* may have genetic height limitations.

## Conclusions

The expansion of tall shrubs onto tundra in the Alaska's arctic National Parks (ARCN) is likely to progress as climate warming continues, but increases will be limited to certain habitats. Tall shrubs in the study area show distinct lower limits for July mean temperature that range from 10.5 to 12.4°C for various species. Additional July warming of 2°C would increase the area ARCN with temperatures suitable for multiple tall shrub species from 23% to 70%. However, soil limitations would restrict tall shrub growth to mainly the drier and less acid soils, which cover less than half of this new area. Alder is likely to be an important species in future expansion, owing to its tolerance of a wide range of soil conditions and the fact that currently much of the study area has mean July temperatures just below its temperature limit. Thus the coming decades are likely to show both striking expansion of tall shrub thickets on suitable sites and—where soils are unsuitable, summer temperatures still too cold, or winters too windy—persistence of vegetation much like today's.

## Supporting Information

S1 Table Shrub canopy volume by species and environmental data. Tab-delimited text format. Shrub canopy volume ("canvol") in m. Mean July temperate, °C ("jultemp"). Ordinal date of spring snow cover loss ("snowday"). Soil pH ("ph"). Soil thaw depth class ("thaw"). Soil drainage class ("drain"). Flooding ("flood"; 0 –none, 1 –yes). Location identifiers ("node", "plot"). Coordinates, decimal degrees WGS1984 datum latitude ("lat") and longitude ("lon"). Elevation, m above sea level ("elev").
(TXT)

S2 Table Shrub canopy volume and environmental data. Tab-delimited text format. Shrub canopy volume for all tall shrubs species combined. For column definitions see S1 Table.
(TXT)
